# Differential Effects of Cannabidiol and Cannabigerol on Cognition, Neuroinflammation, and Blood–Brain Barrier Integrity in a Rat Model of Iron Overload

**DOI:** 10.1007/s12640-026-00826-x

**Published:** 2026-08-28

**Authors:** Layza Kowalski, Anna C. M. Colucci, Juliana B. dos Santos, Beatriz W. Vuaden, Alice L. Herlinger, Jaime E. C. Hallak, Antônio W. Zuardi, José A. S. Crippa, Maria N. M. de Lima, Nadja Schröder

**Affiliations:** 1 Laboratory of Memory Dysfunctions, Department of Physiology, Institute for Basic Health Sciences, Federal University of Rio Grande do Sul, Porto Alegre, Brazil; 2 National Institute of Science and Technology for Translational Medicine (INCT-TM), Conselho Nacional de Desenvolvimento Científico e Tecnológico (CNPq), Brasília, Brazil; 3 Department of Neuroscience and Behavioral Sciences, Ribeirão Preto Medical School, University of São Paulo, São Paulo, Brazil; 4 Laboratory of Psychiatric Neuroimaging (LIM21), Ribeirão Preto Medical School, Hospital das Clínicas HCFMUSP, University of São Paulo, São Paulo, Brazil

**Keywords:** Iron overload, Cognitive decline, Neurodegenerative diseases, Cannabinoids, Neuroprotection

## Abstract

Iron is an essential micronutrient for brain development, participating in mitochondrial respiration, myelination, and neurotransmitter synthesis. However, previous studies have demonstrated that excessive iron during early postnatal life induces oxidative reactions, leading to mitochondrial dysfunction and synaptic failure. These alterations compromise energy metabolism and neuronal integrity, contributing to long-lasting cognitive dysfunction and increased brain vulnerability later in life. This study evaluated the effects of cannabidiol (CBD) and cannabigerol (CBG) on behavioral, neuroinflammatory, and blood-brain barrier (BBB) outcomes in rats exposed to early-life iron overload. Male Wistar rats received iron carbonyl (30 mg/kg, intragastrically) from postnatal day 12 to 14. At three months of age, they were treated intraperitoneally with CBD, CBG (both at 10 mg/kg), or vehicle for 21 days. Cognitive performance was assessed in the open field and object recognition tasks. We examined hippocampal levels of interleukin-1 beta (IL-1β), interleukin-6 (IL-6), tumor necrosis factor alpha (TNF-α), as proinflammatory markers, and occludin, a protein known to regulate BBB permeability. Iron-exposed animals showed impaired recognition memory, with elevated TNF-α and IL-1β, while CBD reversed memory deficits and reduced IL-1β in iron-treated animals, without affecting TNF-α. CBG restored memory, decreased IL-1β in both iron-treated and controls, and increased TNF-α in controls. Also, iron overload reduced occludin expression in vehicle-treated rats which was reversed by both CBD and CBG. These findings highlight inflammation and BBB disruption as mediators of iron-induced cognitive dysfunction and show that both phytocannabinoids act through distinct but complementary mechanisms, supporting their therapeutic potential in neuroinflammation linked to iron overload.

**Supplementary Information:**

The online version contains supplementary material available at https://doi.org/10.1007/s12640-026-00826-x.

## Introduction

Iron plays a vital role in the body and is an essential micronutrient for the central nervous system (CNS), respiratory chain, neurotransmitter synthesis, and myelination. However, iron redox activity between ferrous (Fe²⁺) and ferric (Fe³⁺) forms, promotes the production of hydroxyl radicals through the Fenton reaction (Papanikolaou and Pantopoulos 2005; Ward et al. 2014). These radicals promote oxidative stress (OS), inducing lipid peroxidation, mitochondrial dysfunction, and DNA/protein damage. With aging, iron accumulates in brain regions vulnerable to neurodegenerative diseases (NDs), creating a favorable environment to neuronal degeneration. Excess iron is now considered a key contributor to the onset and progression of NDs such as Alzheimer’s and Parkinson’s (Kenkhuis et al. 2021). Moreover, accumulating evidence suggests an important association of iron deposition in brain regions and cognitive impairment observed in a variety of age-associated NDs (Zhi et al. 2024; Lee et al. 2025).

Neuroinflammation and OS are recognized as key contributors to these conditions, with disruption of the blood–brain barrier (BBB) playing a central role. Under normal conditions, the BBB tightly regulates the exchange of molecules between blood and neural tissue through tight junction (TJ) and adherens junction (AJ) proteins such as occludin, claudins, and zonula occludens-1 (ZO-1) (Rosenblum and Kosman 2022). Inflammation reduces TJ protein expression and compromises barrier function (Rochfort et al. 2016). A weakened BBB not only allows greater iron entry into the brain but also increases exposure to circulating inflammatory mediators, amplifying neurotoxicity and accelerating cognitive decline (Urrutia et al. 2021).

Our research group has long employed a neonatal iron overload model in rats, in which iron is administered orally during three days in the second postnatal week, a period marked by intense neurogenesis and high transferrin receptor expression in the brain (Schröder et al. 2013; Ward et al. 2014). Moreover, the neonatal period represents a critical developmental window characterized by increased BBB permeability to iron and consequent elevated CNS iron uptake, allowing transient iron overload during the first postnatal weeks to induce long-lasting neurobiological consequences. This model reproduces key aspects of iron-induced neurotoxicity observed in aging and NDs, including persistent memory deficits, mitochondrial dysfunction, OS, and glial activation (de Lima et al. 2005; da Silva et al. 2014). Given its cumulative nature, it also provides a robust platform for testing potential neuroprotective strategies.

Cannabidiol (CBD), the main non-psychoactive phytocannabinoid of *Cannabis sativa*, has been extensively studied for its neuroprotective effects, including attenuation of iron-induced memory deficits, restoration of synaptic proteins and reduction of apoptotic signaling in iron-treated rats (Fagherazzi et al. 2012; da Silva et al. 2014; Silva et al. 2018a). More recently, interest has turned to cannabigerol (CBG) because of its antioxidant and anti-inflammatory effects, promoting cell survival and neuroprotection (De Petrocellis et al. 2011; Gugliandolo et al. 2018; Stone et al. 2021), but it was never tested under iron overload conditions.

This study aimed to compare the pharmacological effects of both phytocannabinoids, CBD and CBG, on cognitive function and neuroinflammation in animals subjected to neonatal iron overload, which induces memory deficits similar to those observed in NDs (Schröder et al. 2013).

## Materials and Methods

### Study Design

The study followed a 2 × 3 factorial design with two neonatal treatments (Vehicle [Sorbitol] or Iron) and three adult treatments (Vehicle, CBD, or CBG). In total, 63 male Wistar rats were utilized and randomized into six groups: Sorbitol–Vehicle (*n* = 8), Sorbitol–CBD (*n* = 10), Sorbitol–CBG (*n* = 12); Iron–Vehicle (*n* = 10), Iron–CBD (*n* = 12), and Iron–CBG (*n* = 11). Neonatal treatments were assigned at the litter level, whereas individual animals served as the experimental unit for adult treatments and outcome measures. To avoid any possible litter effect, each experimental group was formed by animals from 3 to 4 different litters. The Sorbitol–Vehicle group served as the control condition.

### Outcome Measures

Primary outcomes were cognitive performance in the Object Recognition Task and hippocampal cytokine levels, such as interleukin-1 beta (IL-1β), interleukin-6 (IL-6) and tumor necrosis factor alpha (TNF-α). BBB integrity was evaluated through occludin expression by Western blot. Consistent with prior work in this model, behavioral and cytokine outcomes were the main focus of this exploratory study.

### Animals

This study was conducted at the Center for Laboratory Animal Reproduction and Experimentation (CREAL) at the Federal University of Rio Grande do Sul (UFRGS), Porto Alegre, RS, Brazil, following approval from the Ethics Committee on Animal Use (CEUA-UFRGS), protocol #45,127. All procedures complied with the guidelines of the Brazilian Guidelines for the Care and Use of Animals in Research and Teaching (CONCEA/MCTI, Resolutions no. 55/2022 and 57/2022), as well as recommendations from the Brazilian Society for Neuroscience and Behavior (SBNeC) and the National Institutes of Health Guide for the Care and Use of Laboratory Animals (8th Edition, 2011), and the ARRIVE guidelines for reporting animal research.

Animals were obtained from the CREAL–UFRGS specific-pathogen-free colony. Pregnant Wistar (*Rattus norvegicus*) dams were acquired at late gestation, and litters were standardized within 48 h of birth. Pups were weaned at postnatal day (PND) 21 and housed until adulthood (PND 90) under controlled temperature (22 ± 1 °C) and a 12:12 h light–dark cycle, with ad libitum access to food and water. Animals were kept in groups of 3–4 animals in individually ventilated cages with sawdust bedding and enrichment items (paper, tissue rolls, plastic blocks), refreshed at each cleaning.

Animals were monitored daily for general health, pain, distress, or abnormal behavior. Manipulations were minimally invasive and performed using gentle handling and appropriate needle sizes to reduce discomfort. No animals reached humane endpoints, and no adverse events occurred.

### Randomization and Blinding

Neonatal treatments were assigned at the litter level using simple randomization (random-number generator). After weaning, litters were randomly allocated to the adult treatment groups (Vehicle, CBD, CBG).

Experimenters responsible for behavioral testing, molecular analyses, and data quantification were blinded to group allocations. Cage positions were rotated weekly to minimize location-related variability. All animal manipulation and behavioral testing occurred during a fixed period of the light phase.

### Sample Size

Sample size was decided based on previous studies published from our research group and others. Usually, behavioral tasks, such as those used in the present study, require a sample size between 8 and 15 animals in each group. The use of this sample size allows consistent and reproducible results for behavioral analysis (de Lima et al. 2005; Fagherazzi et al. 2012; da Silva et al. 2012; Figueiredo et al. 2016). Based on previously published research by our group, for biochemical analysis, including protein analysis using Enzyme-Linked Immunosorbent Assay (ELISA) and western blot, we used sample sizes varying from 4 to 6 animals in each group (Figueiredo et al. 2016; da Silva et al. 2018a; b; Machado et al. 2019). No inclusion or exclusion criteria were established a priori. No exclusions of animals or data points were performed. Because both the iron-overload model and CBD treatment were previously standardized in males, the aim was to compare CBG under equivalent conditions.

### Pharmacological Treatment, Behavioral Tests, and Experimental Design

On PND 12–14 (body weight varying from 20 to 35 g), male pups received a once-daily intragastric administration of either vehicle (5% sorbitol in water) or iron carbonyl (30 mg/kg; ≥97% Fe basis, powder form; Sigma-Aldrich, product number C3518, CAS 7439-89-6), following established protocols (Fagherazzi et al. 2012). This experimental model mimics early-life iron supplementation in humans, applied in a timepoint that coincides with a critical developmental window characterized by high brain iron uptake during the first two postnatal weeks (Schröder et al. 2001, 2013; Ward et al. 2014). Previous studies using this model have demonstrated that neonatal iron overload induces persistent neurobiological alterations, including OS and long-term cognitive deficits that extend into adulthood (de Lima et al. 2005; Schröder et al. 2013).

At three months of age (body weight ranging from 350 to 400 g), rats were randomly assigned to receive daily intraperitoneal injections of vehicle (Tween-80/saline, 1:16), CBD (10 mg/kg), or CBG (10 mg/kg) for 21 consecutive days. This time point was selected to target established and persistent neurobiological alterations induced by early-life iron exposure, which are already evident in young adulthood, while still representing a window in which therapeutic intervention may be effective. Both CBD and CBG were administered as a crystalline isolate (≥ 99% purity; PurMed Global, USA). Certificates of Analysis (CoAs) provided by the supplier are available as Supplementary Material S1 (CBD) and Supplementary Material S2 (CBG). The CBG CoA confirmed > 99.9% CBG purity, non-detectable levels of CBD and Δ9-THC, such as the absence of detectable pesticide residues and residual solvents. The CBD CoA also confirmed the purity specifications and phytocannabinoid profile of the material supplied.

The 21-day treatment period was selected to enable sustained modulation of molecular processes underlying iron-induced cognitive deficits, rather than transient or acute effects. This timeframe, together with the selected CBD dose, has previously been shown to be sufficient to reverse behavioral and molecular alterations in this model (Fagherazzi et al. 2012; da Silva et al. 2014). The CBG dose was chosen based on evidence demonstrating its neuroprotective effects in experimental models of neurodegeneration (Valdeolivas et al. 2015; Gugliandolo et al. 2018; Stone et al. 2021).

Behavioral testing occurred from the 14th to the 16th day after the beginning of pharmacological treatments in adult life. Behavioral assessments were conducted prior to the final experimental endpoint to evaluate functional outcomes under ongoing and stable treatment conditions. The open-field test was performed 24 h before the training phase of the Object Recognition Task and served as habituation to the apparatus (Fagherazzi et al. 2012; Figueiredo et al. 2016). Before testing, animals were allowed to habituate to the experimental environment. The arena consisted of a plywood box (45 × 40 × 60 cm) with a glass front wall. The floor was divided into 12 equal squares to allow quantification of the locomotor activity, and the central zone was defined as the inner area corresponding to approximately 25% of the total arena surface. Each animal was placed individually in the same spot of the arena and allowed to explore freely for 5 min. Locomotion and exploratory parameters (latency to start locomotion, number of line crossings, rearing frequency, and time spent in the center zone) were recorded and later analyzed by a blinded experimenter, using manual counters and stopwatches.

In the Object Recognition Task, rats explored two identical objects (A1 and A2) for 5 min. Long-term memory (LTM) was assessed 24 h later by replacing one object with a novel object (B). Object placement, spacing, and cleaning procedures followed standardized methods (Fagherazzi et al. 2012). Exploration was defined as sniffing or touching with nose or forepaws. All behavioral sessions were videotaped and subsequently analyzed by an experimenter blinded to animals’ experimental condition, using two stopwatches to quantify exploration times, and the recognition index was calculated as TB/(TA + TB), where: TA = time spent exploring the familiar object (A), and TB = time spent exploring the new object.

**Fig. 1:**
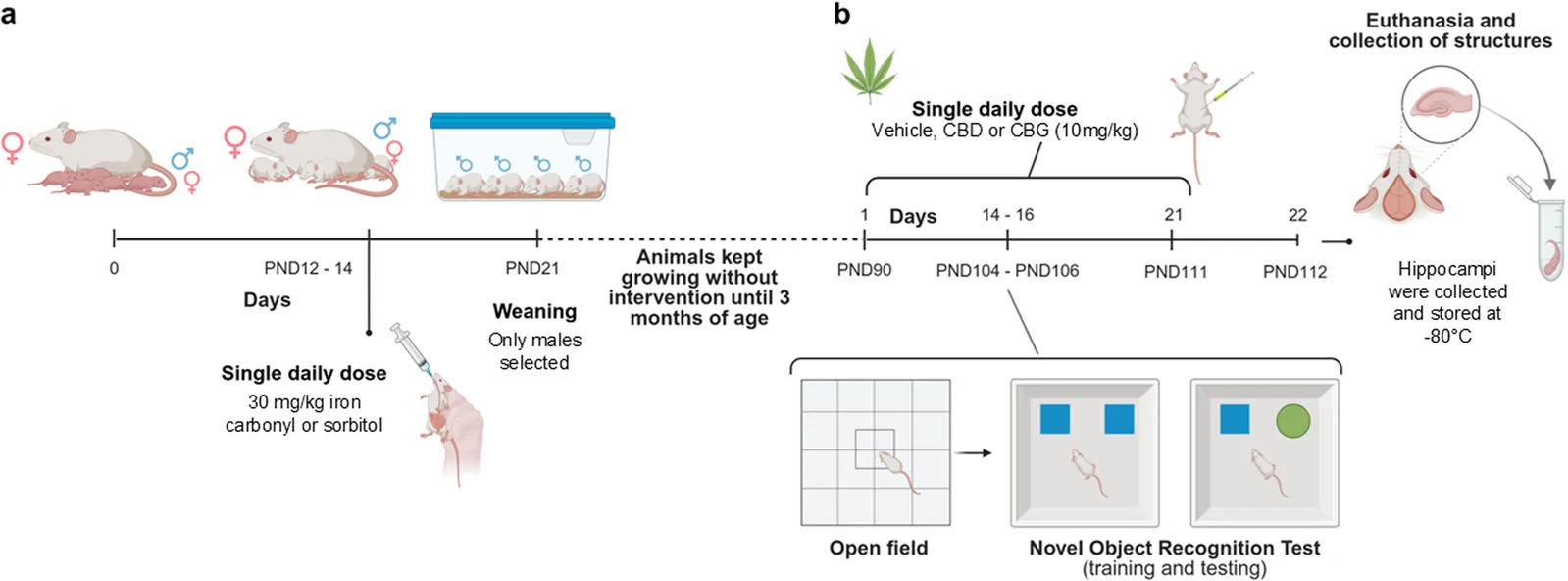
The in vivo experimental design used in the study is represented as a timeline. (**a**) Male pups (*n* = 63) from randomly distributed litters received intragastric administration of iron carbonyl (30 mg/kg/day) or vehicle (5% sorbitol in water) from postnatal days 12 to 14. After treatment, males were maintained until adulthood (3 months old), at which point they were further divided into 6 experimental groups. (**b**) Animals received intraperitoneal (i.p.) administration of cannabidiol (CBD 10 mg/kg/day), cannabigerol (CBG 10 mg/kg/day), or vehicle (VEH - saline with Tween 80) for 21 consecutive days (P90-P111). Behavioral evaluation included the open field and object recognition tasks (P104-P106). After completion of pharmacological treatment, the animals were euthanized, and the hippocampi were collected and stored at −80 °C for subsequent molecular analyses

Molecular analyses were performed at the end of the treatment period to capture cumulative effects of chronic phytocannabinoid administration. Twenty-four hours after the final injection, rats were euthanized by decapitation in a separate room to minimize stress. Hippocampi were rapidly dissected, frozen, and stored at − 80 °C for ELISA and Western blot analyses. The overall experimental timeline is illustrated in Fig. 1.

### ELISA

To analyze the expression of inflammatory cytokines IL-1β, IL-6, and TNF-α, commercial ELISA kits (BMS630, ERA31RB, and KRC3011, respectively, Invitrogen, Thermo Fisher Scientific, USA) were used. Hippocampi from randomly selected animals from each group were weighed and homogenized in 1X cell extraction buffer (50 mM Tris-HCl, pH 7.3; 0.9% NaCl; 0.1% SDS), supplemented with a protease inhibitor cocktail (SigmaFast S8820, Sigma-Aldrich, USA), at a ratio of 90 µL buffer per 10 mg of tissue. Homogenization was performed on ice using a mechanical homogenizer to ensure complete tissue lysis. After 30 min of incubation on ice, the homogenates were centrifuged at 13,500 rpm for 10 min at 4 °C. The supernatant was collected and stored at −20 °C until use (da Silva et al. 2018a). Protein concentrations were quantified using the Bradford method.

For the ELISA assays, 100 µL aliquots of samples, standards, and blanks were plated in duplicate. Absorbance readings were taken at 450 nm using a Zenyth 220RT microplate reader (Biochrom Ltd., UK). Cytokine concentrations were calculated from standard curves generated using the MyCurveFit online tool, applying four-parameter logistic (4PL) interpolation. Results were expressed as picograms per milligram (pg/mg) of protein.

### Western Blot

To assess occludin protein expression, Western blot analysis was performed. Hippocampi from randomly selected animals from each group were weighed and homogenized in lysis buffer (10 mM Tris-HCl, pH 8.0; 1 mM EDTA; 100 mM NaCl; 0.5% Triton X-100; 0.1% SDS), supplemented with protease inhibitor tablets (SigmaFast S8820, Sigma-Aldrich, US), using a ratio of 10 µL buffer per mg of tissue. Homogenization was carried out on ice with a mechanical homogenizer, followed by 30 min of incubation on ice and centrifugation at 13,500 rpm for 10 min at 4 °C. The resulting supernatant was collected, and total protein concentration was determined using the Bradford method.

Protein samples (40 µg/lane) were separated on 10% SDS–polyacrylamide gels and transferred onto nitrocellulose membranes (Bio-Rad, USA). Membranes were blocked with 3% BSA (#A7030-50G, Thermo Fisher Scientific) in Tris-buffered saline containing 0.1% Tween-20 (TBST) for 1 h at room temperature, then incubated overnight at 4 °C with primary antibodies diluted in TBST: anti-occludin (1:200, Thermo Fisher Scientific, #40–4700) and anti-GAPDH (1:500, Cell Signaling Technology, #8884S). On the next day, the membranes were washed and incubated for 1 h at room temperature with HRP-conjugated goat anti-rabbit secondary antibody (1:5000, Abcam, #ab97051). Immunoreactive bands were detected using the ECL Substrate Kit (High Sensitivity, Abcam, #ab133406) and imaged with the iBright FL1000 Imaging System (Thermo Fisher Scientific, US). Band intensity was quantified using ImageJ software and normalized to GAPDH levels.

Bands’ molecular weight was confirmed using two types of ladders: SuperSignal™ Molecular Weight Protein Ladder (Thermo Fisher Scientific, #84785) and Precision Plus Protein™ Kaleidoscope™ Standards (Bio-Rad, #1610375).

### Statistical Analysis

Statistical analyses were performed using SPSS v27.0 (IBM, NY, USA) and a significance level of *p* < 0.05 was adopted. Data normality was assessed using the Shapiro-Wilk test, while homogeneity of variances was verified using Levene’s test. These assumptions guided the selection of the most appropriate statistical tests. All graphs were generated using GraphPad Prism^®^ v8.4.3 (GraphPad Software, MA, USA).

In the open field test, behavioral variables were analyzed using Generalized Linear Models (GLM) according to the distribution of each dataset. The variables “number of crossings” and “number of rearings” exhibited normal and homogeneous distributions, representing count data, which are better modeled by discrete probability distributions. Therefore, these variables were analyzed using a Poisson GLM with a log link function (Pearson’s Chi-square goodness-of-fit; Wald statistics), with neonatal treatment (vehicle or iron) and adult treatment (vehicle, CBD, or CBG) as between-subject factors. Post hoc pairwise comparisons were based on estimated marginal means (EMMs) with Sidak correction for multiple comparisons. The variables “latency to initiate locomotion” and “time spent in the center” did not meet normality criteria and exhibited a right-skewed distribution. Consequently, they were analyzed using a Gamma GLM with a log link function (maximum likelihood estimation; Wald statistics), with post hoc comparisons also based on EMMs with Sidak adjustment. The use of GLM, instead of a traditional two-way ANOVA, was chosen because GLM allows for flexible modeling of non-normally distributed data and the use of appropriate link functions for each variable type, ensuring a more accurate and robust estimation of treatment effects.

The recognition index (training and test phase) did not meet normality assumptions and was analyzed using a GLM with a Gamma distribution and log link function, including neonatal treatment, adult treatment, and their interaction as factors. Post hoc pairwise comparisons were based on EMMs with Sidak correction for multiple comparisons.

The TNF-α level was examined with GLM because Levene’s test indicated violations of variance homogeneity for both main factors. Post hoc pairwise comparisons were based on EMMs with Sidak correction for multiple comparisons. Similarly, normalized levels of IL-1β did not meet the assumptions of normality and were also analyzed using GLM with the same factors and EMM-based Sidak-corrected pairwise comparisons. In contrast, IL-6 data met all statistical assumptions, allowing analysis via two-way ANOVA with Tukey’s post hoc test for multiple comparisons.

Occludin expression data did not meet the assumption of homogeneity of variances, as indicated by Levene’s test, and were therefore analyzed using GLM, including neonatal treatment, adult treatment, and their interaction as fixed factors. Post hoc pairwise comparisons were based on EMMs with Sidak adjustment for multiple comparisons.

## Results

### Behavioral Tests

#### Open Field Test

**Fig. 2:**
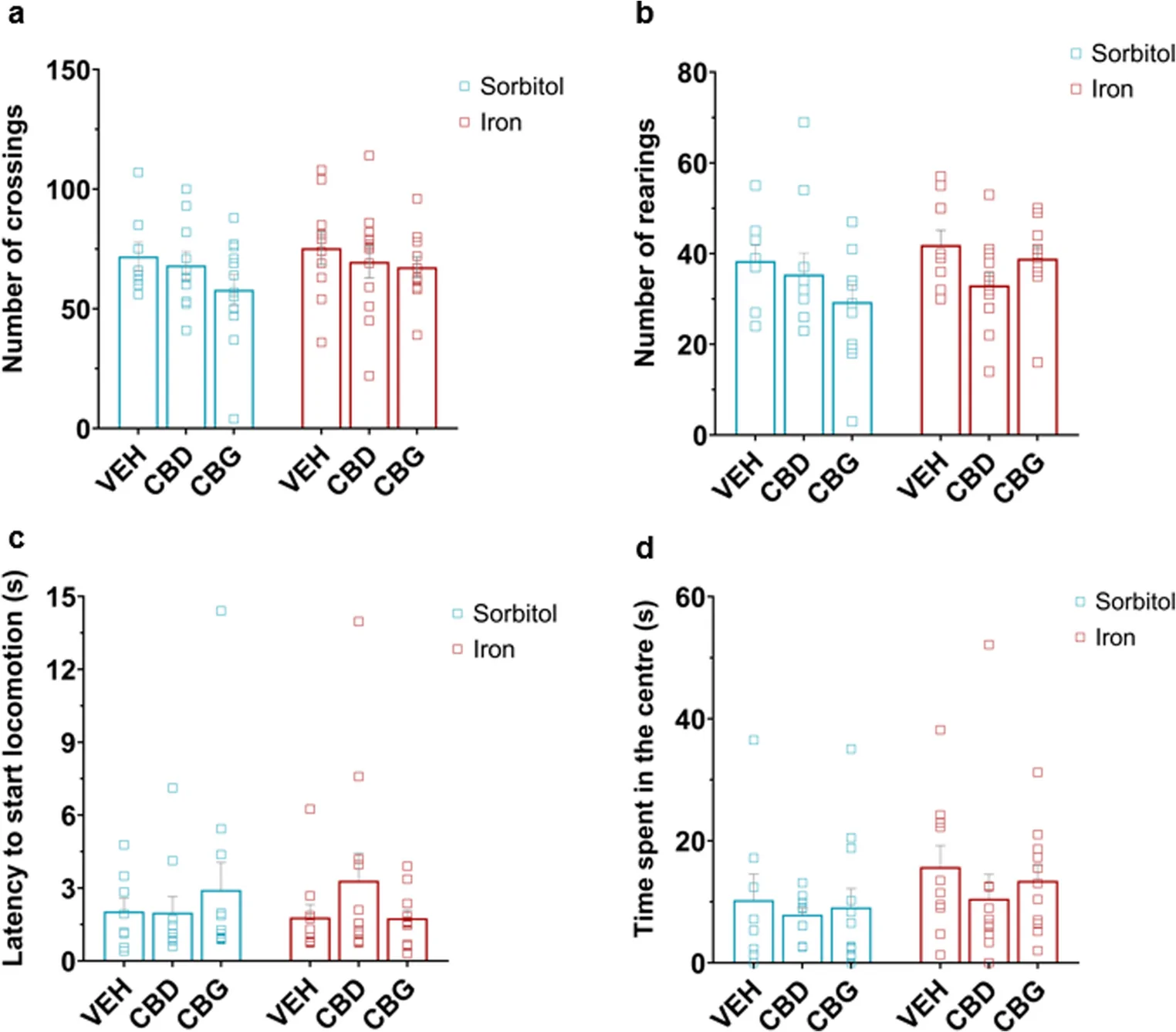
Behavioral parameters in the open field test. Graphs represent the behavioral performance in the open field of adult Wistar rats treated neonatally with Sorbitol or Iron, followed in adulthood by treatment with Vehicle, CBD, or CBG. Group sizes were as follows: Sorbitol–Vehicle (*n* = 8), Sorbitol–CBD (*n* = 10), Sorbitol–CBG (*n* = 12); Iron–Vehicle (*n* = 10), Iron–CBD (*n* = 12), Iron–CBG (*n* = 11). Analyses included: (**a**) number of crossings (horizontal locomotion; GLM, Poisson log-linear model); (**b**) number of rearings (vertical exploration; GLM, Poisson log-linear model); (**c**) latency to initiate locomotion (GLM, Gamma distribution, log link); and (**d**) time spent in the center of the arena (GLM, Gamma distribution, log link). Data are presented as mean ± SEM with individual data points. Statistical comparisons and post hoc analyses were based on estimated marginal means (EMMs). Parametric (a and b) and non-parametric (c and d) variables did not reach statistical significance in the open field analysis (*p* > 0.05 for all factors)

To control for possible alterations in locomotor and exploratory behavior induced by neonatal iron or adult phytocannabinoid treatments, which could interfere with memory acquisition, we performed an analysis of open-field behavior 24 h before the object recognition training session (Fig. 2).

Regarding the number of crossings (Fig. 2a), the GLM revealed no significant main effects of neonatal treatment [Wald χ²(1) = 0.941, *p* = 0.332] or adult treatment [Wald χ²(2) = 3.114, *p* = 0.211], and no significant interaction between the two factors [Wald χ²(2) = 0.569, *p* = 0.752].

Similarly, rearing behavior (vertical exploratory activity, Fig. 2b) showed no effect for the neonatal factor [Wald χ²(1) = 1.487, *p* = 0.223] or the adult treatment factor [Wald χ²(2) = 3.554, *p* = 0.169], and no significant interaction between the two factors [Wald χ²(2) = 3.168, *p* = 0.205]. These findings suggest that neither neonatal iron exposure nor phytocannabinoid treatment in adulthood affected spontaneous locomotor activity or exploratory behavior.

Concerning the latency to initiate locomotion (Fig. 2c), GLM analysis revealed no significant main effects of the neonatal treatment [Wald χ²(1) = 0.049, *p* = 0.826] or adult treatment [Wald χ²(2) = 1.331, *p* = 0.514]. Moreover, no significant interaction between neonatal and adult treatments [Wald χ²(2) = 4.589, *p* = 0.101] on the latency to initiate locomotion was found, indicating that neither early-life iron exposure nor the adult administration of phytocannabinoids significantly altered the time required to start spontaneous locomotion.

The time spent in the central zone (Fig. 2d) showed no statistically significant differences between the neonatal treatment groups [Wald χ²(1) = 2.896, *p* = 0.089] or between the adult treatment groups [Wald χ²(2) = 1,383, *p* = 0.501], and also no significant interaction between the two factors [Wald χ²(2) = 0.151, *p* = 0.927], suggesting absence of anxiety-like behavior.

Overall, these findings indicate that locomotor and exploratory behaviors and anxiety were not influenced by either neonatal iron exposure or adult phytocannabinoid treatment, supporting the interpretation that subsequent cognitive assessments are not confounded by alterations in general activity or anxiety-like behavior.

#### Object Recognition Task

Our primary objective was to investigate whether pharmacological treatment with CBD or CBG in adulthood could mitigate memory deficits induced by iron overload during the neonatal period, an experimental model of memory deficits associated with aging and NDs.

**Fig. 3:**
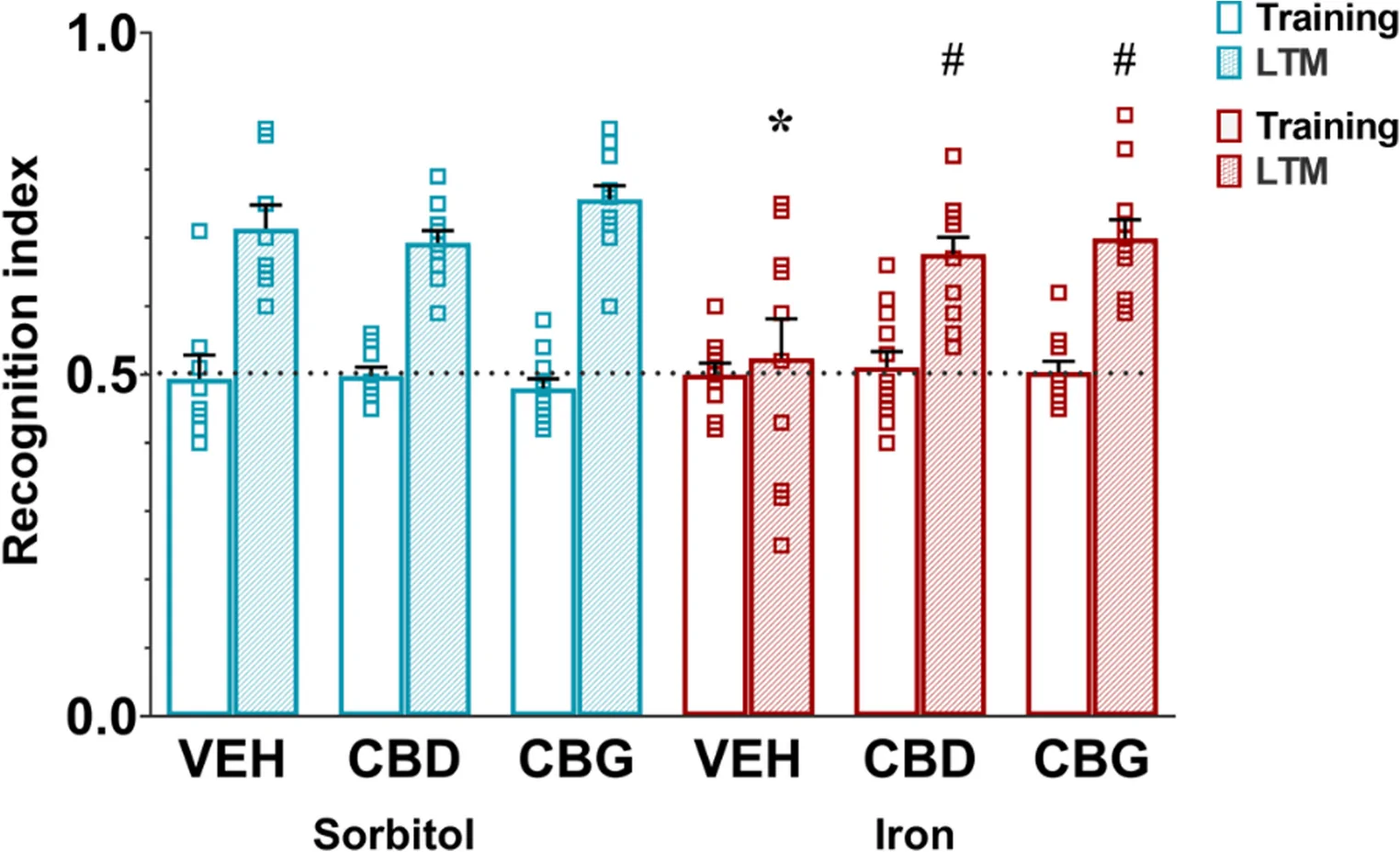
Object recognition task. The bar graphs represent the recognition index data presented as mean ± SEM with individual data points. Adult Wistar rats treated neonatally with Sorbitol or Iron, followed by adult treatment with Vehicle (VEH), cannabidiol (CBD), or cannabigerol (CBG). Object recognition performance expressed as training session and long-term memory (LTM) retention. Group sizes were: Sorbitol–Vehicle (*n* = 8), Sorbitol–CBD (*n* = 10), Sorbitol–CBG (*n* = 12); Iron–Vehicle (*n* = 10), Iron–CBD (*n* = 12), Iron–CBG (*n* = 11). No differences were observed in the training session. In contrast, the test session (LTM) revealed significant main effects of neonatal treatment (*p* = 0.002), adult treatment (*p* = 0.006), and their interaction (*p* = 0.026). Cognitive deficits were induced by neonatal iron overload, as evidenced by the lowest recognition index in the Iron–Vehicle group when compared to the Sorbitol–Vehicle group (*p* = 0.004). Treatment with CBD (*p* = 0.008) and CBG (*p* = 0.002) significantly improved performance in iron-exposed animals, restoring levels comparable to those of the Iron–Vehicle group. Statistical comparisons and post hoc analyses were based on estimated marginal means (EMMs) with Sidak correction. * Indicates significant differences between Iron - Vehicle vs. Sorbitol - Vehicle, *p* < 0.05; # indicates significant differences between Iron - Vehicle vs. Iron – CBD and Iron - CBG, *p* < 0.05

In the recognition index analysis in the training phase (Fig. 3), no effects were observed for the neonatal group [Wald χ²(1) = 0.915, *p* = 0.339], adult group [Wald χ²(2) = 0.527, *p* = 0.768], or their interaction [Wald χ²(2) = 0.240, *p* = 0.887].

The LTM recognition index (from the test session, Fig. 3) demonstrated significant main effects for both neonatal group [Wald χ²(1) = 9.779, *p* = 0.002] and the adult group [Wald χ²(2) = 10.215, *p* = 0.006], as well as their interaction [Wald χ²(2) = 7.329, *p* = 0.026], indicating that the memory-improving effects of adult treatment with CBD or CBG were observed in rats exposed to iron during the neonatal period.

The Iron–Vehicle group displayed the lowest object recognition index among all groups (*p* = 0.004 vs. Sorbitol–Vehicle), confirming that neonatal iron overload impairs recognition memory.

Post hoc analysis revealed that adult treatment with CBD (*p* = 0.008) or CBG (*p* = 0.002) significantly improved recognition memory in iron-exposed rats compared to the Iron–Vehicle group. These findings indicate that both phytocannabinoids exert neuroprotective effects. No significant differences were observed among the Sorbitol-treated groups.

Taken together, these results suggest that neonatal exposure to iron was associated with cognitive damage, and adult intervention with phytocannabinoids had a significant effect on LTM and was able to reverse iron-induced memory impairment.

### Neuroinflammation

To gain a better understanding of the potential mechanisms underlying iron-induced deficits, we investigated the effects of iron overload during the neonatal period on inflammatory parameters in the hippocampus of adult rats. Given the known anti-inflammatory effects of phytocannabinoids, we also aimed to investigate whether their potential in reversing iron-induced memory deficits could be related to counteracting neuroinflammation.

#### TNF-α

**Fig. 4:**
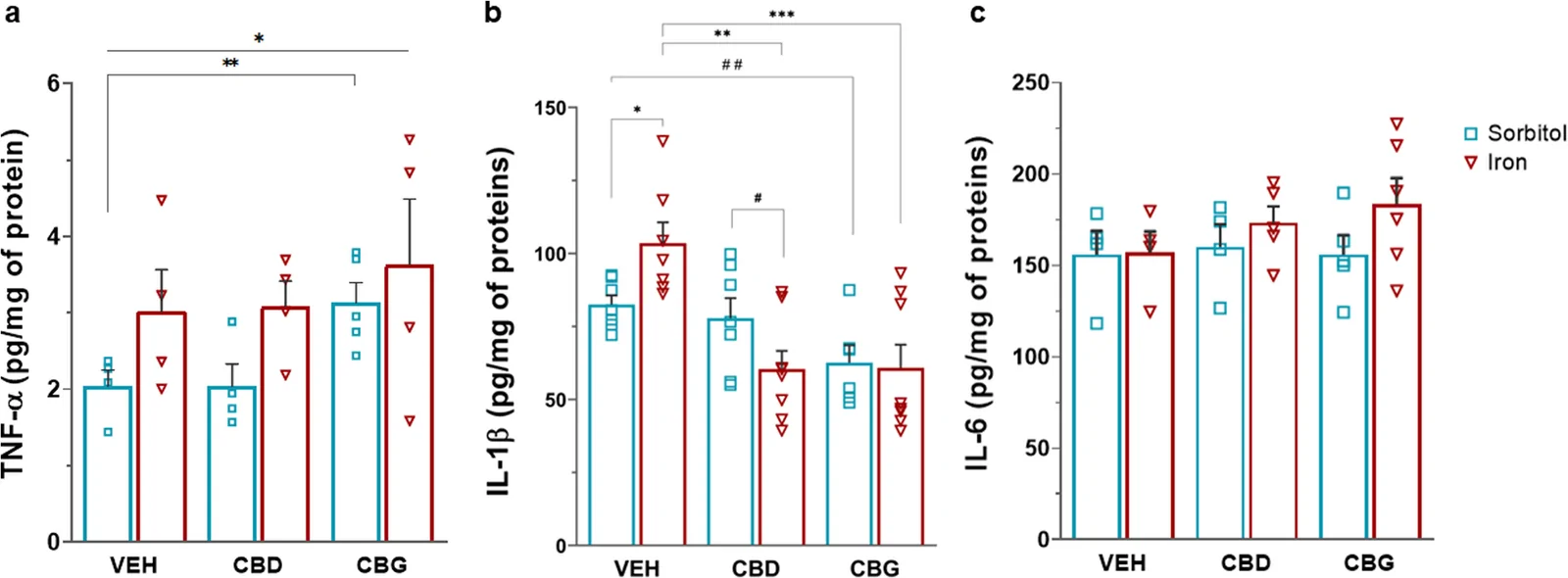
Hippocampal cytokine levels (TNF-α, IL-1β, and IL-6) in adult rats treated with CBD or CBG following neonatal iron overload. (**a**) Tumor Necrosis Factor-α (TNF-α). Bar graphs represent TNF-α concentrations in animals treated neonatally with Sorbitol or Iron and, in adulthood, with Vehicle (VEH), cannabidiol (CBD), or cannabigerol (CBG). Group sizes: Sorbitol–VEH (*n* = 4), Sorbitol–CBD (*n* = 4), Sorbitol–CBG (*n* = 5); Iron–VEH (*n* = 4), Iron–CBD (*n* = 4), Iron–CBG (*n* = 4). Neonatal iron exposure significantly increased TNF-α levels (*p* = 0.011). CBG increased TNF-α under basal conditions (Sorbitol–CBG vs. Sorbitol–VEH, *p* = 0.049), whereas CBD had no effect. * Indicates *p* < 0.05, Sorbitol vs. Iron (main effect); ** indicates *p* < 0.05, Sorbitol–CBG vs. Sorbitol–VEH; (**b**) Interleukin-1β (IL-1β). IL-1β concentrations for the same groups. Group sizes: Sorbitol–VEH (*n* = 7), Sorbitol–CBD (*n* = 7), Sorbitol–CBG (*n* = 6); Iron–VEH (*n* = 7), Iron–CBD (*n* = 8), Iron–CBG (*n* = 8). Adult treatment (*p* < 0.001) and the interaction between neonatal and adult treatments (*p* < 0.05) were significant. Iron–VEH animals showed elevated IL-1β vs. Sorbitol–VEH (*p* = 0.016). Both CBD and CBG reduced IL-1β levels in iron-exposed rats (*p* < 0.001). CBD also reduced IL-1β below Sorbitol–CBD levels (*p* = 0.038), and CBG reduced IL-1β even in controls. * Indicates *p* < 0.05 (Iron–VEH vs. Sorbitol–VEH); ** indicates *p* < 0.001 (Iron–CBD vs. Iron–VEH); *** Indicates *p* < 0.001 (Iron–CBG vs. Iron–VEH); # indicates *p* < 0.05 (Iron–CBD vs. Sorbitol–CBD). ## Indicates *p* < 0.05, Sorbitol–CBG vs. Sorbitol–Vehicle. (**c**) Interleukin-6 (IL-6). IL-6 levels in the same treatment groups. Group sizes: Sorbitol–VEH (*n* = 4), Sorbitol–CBD (*n* = 4), Sorbitol–CBG (*n* = 5); Iron–VEH (*n* = 4), Iron–CBD (*n* = 5), Iron–CBG (*n* = 6). There were no significant effects of neonatal treatment, adult treatment, or their interaction (all *p* > 0.05). Data are presented as mean ± SEM with individual data points. Statistical comparisons for TNF-α and IL-1β were performed using GLM with EMMs and Sidak-corrected post hoc comparisons. IL-6 data were analyzed using two-way ANOVA followed by Tukey’s post hoc test

Statistical analysis of hippocampal TNF-α levels (Fig. 4a) revealed a significant effect of neonatal treatment (Wald χ²(1) = 6.472, *p* = 0.011). Neonatal treatment had a significant main effect on TNF-α levels, indicating an overall increase associated with iron exposure. The adult treatment factor showed a marginally significant effect (Wald χ²(2) = 5.862, *p* = 0.053), while the interaction between factors was not significant (Wald χ²(2) = 0.568, *p* = 0.753), suggesting that phytocannabinoid treatments did not reverse the iron-induced elevation of TNF-α, as further supported by post hoc comparisons.

Pairwise analyses indicated that CBD treatment did not alter TNF-α expression under either condition. In Sorbitol-treated animals, TNF-α levels were nearly identical between the Sorbitol–CBD and Sorbitol–Vehicle groups (*p* = 0.995). Similarly, in Iron-exposed animals, TNF-α concentrations in the Iron–CBD group did not differ from the Iron–Vehicle group (*p* = 0.907). Thus, CBD failed to modulate hippocampal inflammation, suggesting a limited or pathway-specific anti-inflammatory effect under the conditions tested.

CBG showed a different profile. The Iron–CBG group did not differ significantly from the Iron–Vehicle group (*p* = 0.295), indicating no protective effect against iron-induced inflammation. However, in the absence of iron overload, the Sorbitol–CBG group displayed significantly higher TNF-α levels than the Sorbitol–Vehicle group (*p* = 0.049). This result suggests that CBG may even promote a mild pro-inflammatory response under basal conditions, rather than exerting anti-inflammatory effects.

#### IL-1β

The overall model was statistically significant [Omnibus test: χ²(5) = 27.929, *p* < 0.001], confirming the fit of the analysis. Examination of hippocampal IL-1β levels (Fig. 4b) revealed no effect of neonatal treatment alone [Wald χ²(1) = 0.014, *p* = 0.907], while significant effects emerged for adult treatment [Wald χ²(2) = 28.399, *p* < 0.001] and for the interaction between factors [Wald χ²(2) = 10.199, *p* = 0.006]. Pairwise comparisons indicated that the Iron–Vehicle group displayed significantly higher IL-1β concentrations compared to the Sorbitol–Vehicle group (*p* = 0.016), confirming that neonatal iron overload enhanced hippocampal inflammation. CBD significantly reduced IL-1β levels under iron-overload conditions. Animals in the Iron–CBD group had significantly lower IL-1β levels than those in the Iron–Vehicle group (*p* < 0.001). In contrast, CBD did not alter cytokine levels under basal conditions, as the Sorbitol–CBD group and Sorbitol–Vehicle group did not differ significantly (*p* = 0.590). Notably, IL-1β concentrations were even lower in the Iron–CBD group compared with the Sorbitol–CBD group (*p* = 0.038), suggesting that CBD not only counteracted iron-induced inflammation but may also enhance anti-inflammatory signaling beyond baseline levels under injury conditions.

Similarly, CBG treatment reduced IL-1β expression in animals that underwent neonatal iron overload. The Iron–CBG group showed significantly lower cytokine levels than the Iron–Vehicle group (*p* < 0.001). In addition, CBG decreased IL-1β under basal conditions, as the Sorbitol–CBG group exhibited significantly reduced levels compared to the Sorbitol–Vehicle group (*p* = 0.027). Together, these findings highlight a modulatory effect of CBG on IL-1β levels, effective both in the presence and absence of iron-induced hippocampal inflammation.

These findings indicate that CBD and CBG modulate inflammatory responses in a cytokine-specific manner, particularly by reducing IL-1β levels.

This profile supports their potential use as therapeutic tools in neuroinflammatory conditions, and CBG may even have a broader anti-inflammatory profile, functioning even in the absence of an apparent inflammatory trigger.

#### IL-6

Statistical comparisons of IL-6 levels (Fig. 4c) indicated no statistically significant effects. No significant effects of adult group [F(2,18) = 0.322; *p* = 0.729] or neonatal group [F(1,18) = 0.864; *p* = 0.365] were observed, nor interaction between neonatal and adult treatment groups [F(2,18) = 0.258; *p* = 0.776]. The overall model was also non-significant [F(5,18) = 0.404; *p* = 0.839], with low explanatory power (R² = 0.101; adjusted R² = −0.149), suggesting that neither neonatal nor adult treatments had a significant influence on IL-6 levels under the tested conditions.

### BBB Integrity

**Fig. 5:**
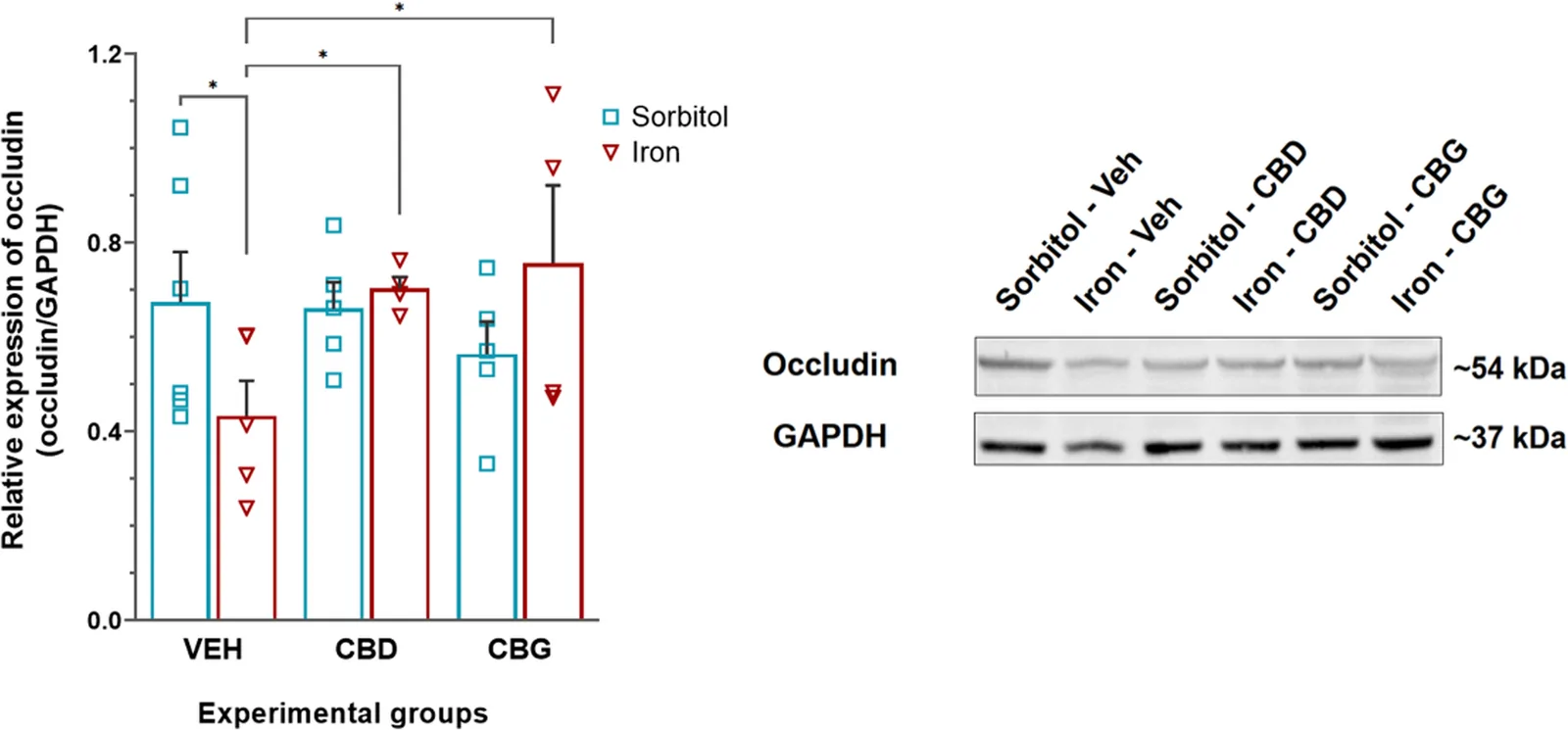
Normalized occludin levels (mean ± SEM) and a representative immunoblot in the hippocampus of adult rats subjected to neonatal treatment with sorbitol or iron, followed by adult treatment with vehicle (VEH), cannabidiol (CBD), or cannabigerol (CBG). Group sizes were as follows: Sorbitol–Vehicle (*n* = 6), Sorbitol–CBD (*n* = 5), Sorbitol–CBG (*n* = 5); Iron–Vehicle (*n* = 5), Iron–CBD (*n* = 4), and Iron–CBG (*n* = 4). A significant interaction was observed between neonatal and adult treatments (*p* = 0.023). Post hoc analysis revealed that neonatal iron overload significantly reduced occludin levels compared to neonatal sorbitol in vehicle-treated animals, and that both CBD and CBG in adulthood significantly increased occludin expression in the iron-exposed groups compared to vehicle treatment. Data are presented as mean ± SEM with individual data points. Statistical comparisons and post hoc analyses were based on estimated marginal means (EMMs) obtained from generalized linear model analysis. * Indicates *p* < 0.05 versus the indicated group

To investigate whether neuroinflammatory condition induced by neonatal iron overload could influence BBB integrity and pharmacological treatment with CBD or CBG in adulthood would be able to counteract a proposed iron-induced BBB disruption, Western blot analysis of occludin was performed and expressed as estimated marginal means from generalized linear model analysis (Fig. 5).

GLM analysis revealed that neither factor alone had a significant effect (adult treatment: Wald χ²(2) = 2.946; *p* = 0.229; neonatal treatment: Wald χ²(1) = 0.001; *p* = 0.975). However, a statistically significant interaction between neonatal and adult treatments was detected [Wald χ²(2) = 7.530; *p* = 0.023].

Pairwise comparisons showed that neonatal iron overload significantly reduced occludin expression compared to controls (*p* = 0.026). In animals exposed to neonatal iron overload, CBD treatment significantly increased occludin levels compared with the iron-vehicle (*p* = 0.025), and CBG treatment had an even stronger effect (*p* = 0.007). No significant differences among adult treatments were observed within the Sorbitol groups.

These results indicate that neonatal iron overload disrupts BBB integrity, and both CBD and CBG treatments in adulthood can restore occludin expression, preserve the barrier and counteract TJ disruption possibly associated with early-life iron-induced neuroinflammation.

## Discussion

Neonatal iron overload is a well-established model of persistent neurobiological alterations. In the present study, it was associated with impaired recognition memory in adulthood, consistent with previous reports of long-term cognitive dysfunction (de Lima et al. 2005; Schröder et al. 2013). Importantly, deficits were specific to memory retention, not related to motor or exploratory changes.

Previous studies indicate that excess iron promotes OS, glial activation, apoptosis, and loss of synaptic proteins, particularly those associated with synaptic integrity and plasticity, such as synaptophysin (de Lima et al. 2005; da Silva et al. 2014). Our findings reinforce this, showing for the first time that neonatal iron exposure induces long-lasting hippocampal alterations while sustaining a pro-inflammatory state, marked by elevated TNF-α and IL-1β. These cytokines knowingly impair synaptic function and memory, indicating neuroinflammation as a key mechanism (Allan et al. lca^2005^; Feng et al. 2017; Khaksar and Bigdeli 2017).

Mechanistically, this response can be attributed to iron’s ability to generate hydroxyl radicals via Fenton chemistry, causing oxidative damage. Also, OS activates microglia and astrocytes, which release proinflammatory mediators and amplify neuronal injury (Kenkhuis et al. 2021). Accordingly, we have previously demonstrated increased reactive astrocytosis, revealed by densitometry of glial fibrillary acidic protein (GFAP) immunoreactive astrocytes, in the hippocampus of iron-treated adult rats when compared to age-matching controls (Fernandez et al. 2011). Thus, the persistent inflammatory alterations may contribute to the cognitive deficits observed after neonatal iron overload, possibly in combination with other mechanisms previously verified, including neuronal and synaptic alterations.

The role of TNF-α in neurodegeneration has been increasingly recognized. In neurons, TNF-α promotes apoptosis, excitotoxicity, and synapse loss (Kenkhuis et al. 2021; Wang et al. 2022). In glial cells, it maintains a self-amplifying inflammatory cycle by stimulating further release of cytokines and ROS (Khaksar and Bigdeli 2017). Consequently, TNF-α emerges as both a mediator and amplifier of iron-induced toxicity. Additionally, it has been demonstrated that persistent elevation of IL-1β impairs long-term potentiation (LTP), reduces dendritic spine density, and interferes with neurogenesis (Allan et al. 2005). These mechanisms might explain the memory deficits, suggesting chronic neuroinflammation as a mediator.

Although CBD is known to inhibit TNF-α production via NF-κB pathway suppression and TNF receptor downregulation (Esposito et al. 2006, 2007; Carrier et al. 2006; Khaksar and Bigdeli 2017), in our study it did not significantly reduce this cytokine in iron-overloaded animals. This lack of effect likely reflects a state of chronic inflammation, where NF-κB signaling becomes persistently activated and less responsive to modulation. Therefore, CBD’s ability to suppress TNF-α appears more effective in acute or transient inflammatory contexts (de Lima et al. 2005; Atalay et al. 2019; Urrutia et al. 2021), rather than long-term, as is the case of the current model.

In contrast, CBD reduced IL-1β levels in iron-exposed animals, even below basal levels. This suggests that other mechanisms, including partial NF-κB inhibition (Esposito et al. 2007; Atalay et al. 2019), 5-HT1A receptor activation, and adenosine uptake (Carrier et al. 2006; Pazos et al. 2013), were sufficient to attenuate IL-1β signaling despite chronic inflammation. These selective effects likely underlie the observed improvement in recognition memory, given IL-1β’s established role in disrupting LTP and synaptic plasticity.

CBG exhibited a markedly distinct pharmacological profile compared with CBD. It significantly increased hippocampal TNF-α levels compared to vehicle controls. This unexpected elevation suggests that CBG and CBD act through different pathways, and that CBG might have dual features, depending on the baseline inflammatory state. Interestingly, CBG displayed a significant effect on IL-1β, by reducing its level in animals exposed to iron while also lowering the cytokine levels under basal conditions. This dual outcome indicates that CBG’s actions are context-dependent (De Petrocellis et al. 2011; Borrelli et al. 2013; Gugliandolo et al. 2018). Mechanistically, the effects of CBG relate to its broad receptor activity. Unlike other phytocannabinoids, it lacks CB1 affinity, has partial CB2 agonism, and indirectly elevates anandamide (AEA) by inhibiting fatty acid amide hydrolase (FAAH), though less effectively than CBD (De Petrocellis et al. 2011; Stone et al. 2021). CBG also engages peroxisome proliferator-activated receptors (PPARs), particularly PPARγ (Hind et al. 2016; Gugliandolo et al. 2018; Stone et al. 2021), and modulates α2-adrenergic and serotonin 5-HT1A receptors, linking it to metabolic, anti-inflammatory, and neuroprotective functions in the CNS (Mendiguren et al. 2023).

This pharmacological profile helps explain the contrasting outcomes observed and their association with neuroprotective effects (Valdeolivas et al. 2015; di Giacomo et al. 2020; Stone et al. 2021). In control animals, where there is no significant inflammatory challenge, activation of α2-adrenergic and serotonergic pathways may shift signaling toward a mild pro-inflammatory state, consistent with the increase in TNF-α. In contrast, under iron-induced neuroinflammation, CBG’s ability to engage PPARγ, modulate CB1 through AEA elevation, and activate protective TRP pathways appears to dominate, leading to a reduction in IL-1β and improved cognitive performance. The absence of an effect on TNF-α in the iron group suggests that this cytokine may be less sensitive to the mechanisms activated by CBG.

No changes were observed in hippocampal IL-6 levels after neonatal iron overload. It is possible that the peak of IL-6 expression was not captured in this study or that a stronger stimulus is required for its detection. As previous studies indicate, IL-6 often shows transient fluctuations, particularly under phytocannabinoid influence (De Petrocellis et al. 2011; Gugliandolo et al. 2018; di Giacomo et al. 2020). Thus, the absence of alterations here suggests that IL-6 is unlikely to be a useful neuroinflammatory marker in the neonatal iron overload model.

Collectively, our findings show that phytocannabinoids might modulate hippocampal inflammation in a cytokine-specific way. Both CBD and CBG reduced IL-1β under iron-induced neuroinflammation and even in baseline conditions, but neither reversed TNF-α increases. Thus, IL-1β emerges as a more sensitive target, while TNF-α may require stronger or additional stimuli for modulation. These results are particularly relevant given the major role of neuroinflammation in hippocampal vulnerability and memory impairment. Elevated IL-1β and TNF-α are central to processes associated with learning and memory impairment, while reducing inflammatory signaling can restore cognitive function (Allan et al. 2005; Feng et al. 2017). However, additional mechanisms previously associated with iron overload, including neuronal damage and synaptic dysfunction, may also contribute to the observed memory impairment.

Loss of BBB integrity is a well-known consequence of OS and neuroinflammation (Rochfort et al. 2016; Rosenblum and Kosman 2022), and our results extend this precept to neonatal iron overload. Excess iron promotes ROS generation and ferroptosis, releasing redox-active iron that may perpetuate cellular and vascular damage (Zhao et al. 2023). Previous studies have shown that matrix metalloproteinases (MMPs), induced under OS conditions, can degrade basal lamina components and TJ proteins (Rosenberg et al. 1995; Rosenberg 2002), potentially contributing to the reduction in occludin expression observed in the present study. In addition, inflammatory cytokines have been reported to downregulate TJ proteins and impair BBB-associated pathways, increasing barrier permeability (Rochfort et al. 2016). Thus, early-life iron exposure may induce persistent alterations in proteins associated with BBB structural organization, which could then contribute to long-term neurobiological dysfunction.

CBD and CBG treatments in adulthood reversed the reduction of occludin expression in animals exposed to neonatal iron overload. These findings suggest that phytocannabinoids may modulate molecular components associated with BBB structure and protect its integrity under iron-overload conditions. Previous studies support a potential BBB-related effect of CBD through modulation of inflammatory and oxidative pathways. In traumatic brain injury, CBD reduced TNF-α and IL-1β while increasing TJs proteins (Jiang et al. 2021). Similar effects were seen in cerebral ischemia, where CBD reduced TNF-α, TNFR1, and NF-κB signaling, accompanied by decreased BBB permeability (Khaksar and Bigdeli 2017). After intraventricular hemorrhage, CBD decreased MMPs activity and partially restored occludin expression which was associated with NF-κB inhibition and TNF-α reduction (Pozo et al. 2024). Together, these findings from previous studies are in line with our results and suggest the possible involvement of inflammatory and BBB-related pathways in the effects observed after phytocannabinoid treatment.

Although evidence is still limited, CBG appears to be a promising neuroprotective agent for BBB integrity. In vitro studies with human BBB cells showed that CBG reduced pro-inflammatory markers and proteins linked to DNA damage, such as p53, which contributes to ferroptosis and barrier dysfunction (Stone et al. 2021; Zhao et al. 2023).

Iron accumulation in humans, as seen in conditions such as NDs, has been associated with cognitive impairment and memory deficits (Spence et al. 2020). These findings reinforce the translational relevance of our model, and suggest that phytocannabinoids, may modulate molecular alterations associated with iron-induced neurotoxicity (Fagherazzi et al. 2012; da Silva et al. 2012) in patients as well.

Overall, both CBD and CBG modulated occludin expression after neonatal iron overload, consistent with previously reported neuroprotective and immunomodulatory properties. Previous studies suggest that CBD may influence inflammatory and oxidative pathways associated with TJ regulation, whereas the mechanisms underlying CBG effects remain less understood. Importantly, molecular alterations in inflammatory markers and occludin expression were accompanied by improved recognition memory, supporting the possibility that phytocannabinoids may attenuate persistent neurobiological alterations induced by early-life iron exposure.

This study has some limitations that should be considered when interpreting the findings. First, only male rats were included, and the present results cannot be generalized to females, particularly given the known sex-related differences in neuroinflammatory responses and endocannabinoid signaling. Second, although occludin expression was used as a molecular marker of BBB integrity, no functional assessment of BBB permeability was performed. Therefore, our findings demonstrate restoration of a TJ-associated protein rather than direct recovery of BBB function. Third, the inflammatory analysis was restricted to some cytokines, and additional inflammatory mediators, glial activation markers, OS parameters, and signaling pathways were not investigated. Consequently, the mechanisms underlying the neuroprotective effects of CBD and CBG remain only partially characterized. Therefore, while the present findings support an association between phytocannabinoid treatment, reduced neuroinflammation, restoration of occludin expression, and improved recognition memory, the precise molecular mechanisms underlying these effects remain to be elucidated.

The present study extends previous investigations of neonatal iron overload by providing the first comparative assessment of CBD and CBG in this model. By following the experimental conditions previously used in this model, it was possible to directly compare the present findings with earlier studies from our group. Altogether, our findings support CBG as a promising and yet underexplored therapeutic candidate for neuroinflammation and iron-related brain dysfunction.

## Conclusions

In summary, our findings confirm that neonatal iron overload induces persistent memory impairments, and further show that these deficits are associated with neuroinflammation and increased BBB permeability, as indicated by reduced occludin expression. In line with the primary aim of this study, the comparative evaluation of CBD and CBG demonstrated that both cannabinoids restored occludin expression while attenuating neuroinflammation, changes that were accompanied by improved recognition memory performance. Together, these findings support the hypothesis that modulation of neuroinflammatory responses and BBB integrity contributes to cognitive recovery in adulthood following early-life iron overload and highlight both CBD and CBG as promising therapeutic candidates for iron-related neurological disorders.

## Supplementary Information

Below is the link to the electronic supplementary material.

Supplementary File 1 (PDF 358 KB)

Supplementary File 2 (PDF 1.47 MB)

Supplementary File 3 (PDF 1.38 MB)

## Data Availability

No datasets were generated or analysed during the current study.
