# Supplementary material for: Differential Effects of Cannabidiol and Cannabigerol on Cognition, Neuroinflammation, and Blood–Brain Barrier Integrity in a Rat Model of Iron Overload
Source: Neurotox Res. 2026 Aug 28;44(5):48. doi: 10.1007/s12640-026-00826-x (PMC13524965; doi:10.1007/s12640-026-00826-x)
Supplement: Supplementary file 1 [file 12640_2026_826_MOESM1_ESM.pdf]

Informational Use Only
Not For Regulatory Use

Certificate of Analysis

Juniper Analytics LLC
1334 NE 2nd St
Bend, OR
(541) 382-3796
http://www.juniperanalyticsllc.com
ORELAP: License # 4101-001

Coastal Labs
, OR 97701
(619) 665-9966
Lic. #

Sample: 20JA1726.02

Concentrate/Extract Non Compliance
Residual Solvents-SOL-A, Pesticides-PES-A
and Potency-CAN

Report Date: 10/7/2020
Intake Date: 10/02/2020
Harvest/Prod. Date:

CBG Isolate

Strain: CBG Isolate
METRC Sample: ; METRC Batch #: ;
External Batch #:CBGI006; Analytical Batch #: PO-2020-10-02-01

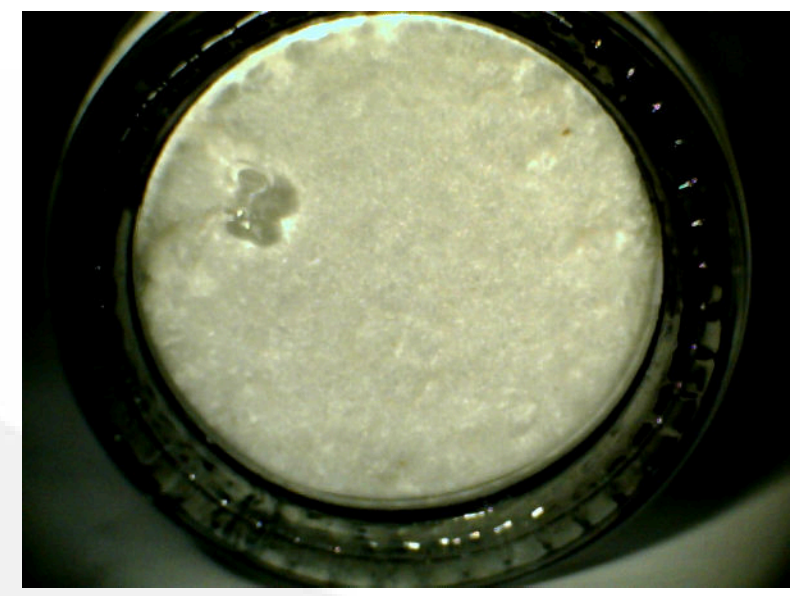

**<LOQ** Total THC %	**<LOQ** Total CBD %
**<LOQ** Total THC mg/g	**<LOQ** Total CBD mg/g

Pesticides	Pass
Water Activity	Not Tested
Moisture Content	Not Tested

See attached sheets for details

This report was amended to adjust CBG values to >99.9% on 10/15/2020.
This product has been tested by Juniper Analytics LLC, using valid testing methodologies and a quality system as required by state law. Values reported relate only to the product tested and batched under the batch number identified above. Juniper Analytics LLC makes no claims as to the efficacy, safety, or other risks associated with any detected or non-detected level of any compounds reported herein. This Certificate must not be altered, and shall not be reproduced except in full, without the written approval of Juniper Analytics LLC.

Ben Armstrong
QA Review

Confident Cannabis
All Rights Reserved
support@confidentcannabis.com
(866) 506-5866
www.confidentcannabis.com

Juniper Analytics LLC
1334 NE 2nd St
Bend, OR
(541) 382-3796
http://www.juniperanalyticsllc.com
ORELAP: License # 4101-001

Coastal Labs

, OR 97701
(619) 665-9966
Lic. #

Sample: 20JA1726.02

Concentrate/Extract Non Compliance
Residual Solvents-SOL-A, Pesticides-PES-A
and Potency-CAN

Report Date: 10/7/2020
Intake Date: 10/02/2020
Harvest/Prod. Date:
Potency Analysis Date: 10/05/2020

CBG Isolate

Strain: CBG Isolate
METRC Sample: ; METRC Batch #: ;
External Batch #:CBGI006; Analytical Batch #: PO-2020-10-02-01

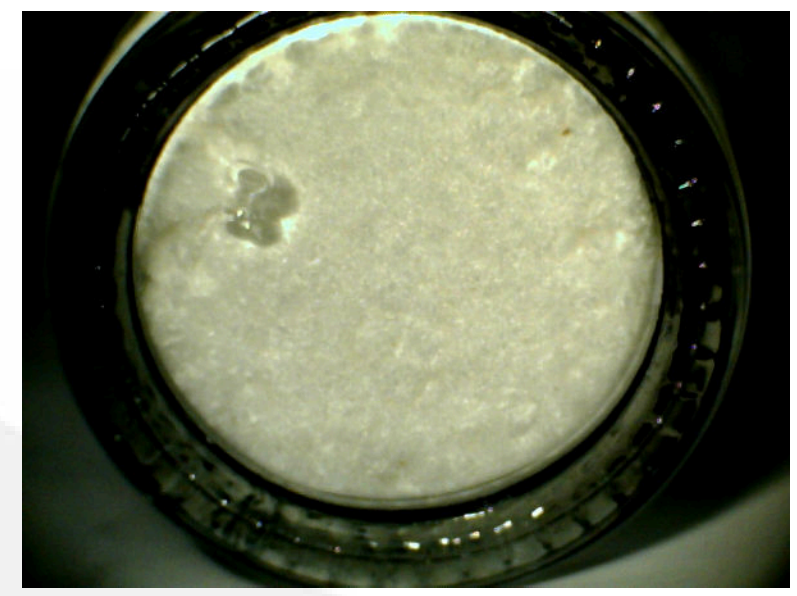

Cannabinoid Profile

<LOQ	<LOQ
Total THC	Total CBD
<LOQ	<LOQ
Total THC mg/g	Total CBD mg/g

Analyte	LOQ	Mass	Mass
	mg/g	%	mg/g
THCa	1.000	<LOQ	<LOQ
Δ9-THC	1.000	<LOQ	<LOQ
Δ8-THC	1.000	<LOQ	<LOQ
THCV	1.000	<LOQ	<LOQ
CBDa	1.000	<LOQ	<LOQ
CBD	1.000	<LOQ	<LOQ
CBN	1.000	<LOQ	<LOQ
CBGa	1.000	<LOQ	<LOQ
CBG	1.000	>99.9000	>999.000
CBC	1.000	<LOQ	<LOQ
**Total**		**>99.9000**	**>999.000**

QC Batch ID: PO-2020-10-02-01
Total THC = THCa * 0.877 + Δ9-THC
Total CBD = CBDa * 0.877 + CBD
Instrument: HPLC/DAD; Method: JA-Potency-Proprietary; LOQ = Limit of Quantitation; The reported result is based on a sample weight with the applicable moisture content for that sample; Unless otherwise stated all quality control samples performed within specifications established by the Laboratory. Potency Analysis (Oregon Compliance Standard OAR 333-007-0430).

Ben Armstrong
QA Review

Confident Cannabis
All Rights Reserved
support@confidentcannabis.com
(866) 506-5866
www.confidentcannabis.com

Juniper Analytics LLC
1334 NE 2nd St
Bend, OR
(541) 382-3796
http://www.juniperanalyticsllc.com
ORELAP: License # 4101-001

Coastal Labs

, OR 97701
(619) 665-9966
Lic. #

Sample: 20JA1726.02

Concentrate/Extract Non Compliance
Residual Solvents-SOL-A, Pesticides-PES-A
and Potency-CAN

Report Date: 10/7/2020
Intake Date: 10/02/2020
Harvest/Prod. Date:
Pesticides Analysis Date: 10/05/2020

CBG Isolate

Strain: CBG Isolate
METRC Sample: ; METRC Batch #: ;
External Batch #:CBGI006 ; Analytical Batch #: Pest-2020-10-02-01

Pesticides

Pass

Analyte	LOQ PPM	Limit PPM	Results PPM	Status
Abamectin	0.25	0.50	<LOQ	Pass
Acephate	0.20	0.40	<LOQ	Pass
Acequinocyl	1.00	2.00	<LOQ	Pass
Acetamiprid	0.10	0.20	<LOQ	Pass
Aldicarb	0.20	0.40	<LOQ	Pass
Azoxystrobin	0.10	0.20	<LOQ	Pass
Bifenazate	0.10	0.20	<LOQ	Pass
Bifenthrin	0.10	0.20	<LOQ	Pass
Boscalid	0.20	0.40	<LOQ	Pass
Carbaryl	0.10	0.20	<LOQ	Pass
Carbofuran	0.10	0.20	<LOQ	Pass
Chlorantraniliprole	0.10	0.20	<LOQ	Pass
Chlorfenapyr	0.50	1.00	<LOQ	Pass
Chlorpyrifos	0.10	0.20	<LOQ	Pass
Clofentezine	0.10	0.20	<LOQ	Pass
Cyfluthrin	0.50	1.00	<LOQ	Pass
Cypermethrin	0.50	1.00	<LOQ	Pass
Daminozide	0.50	1.00	<LOQ	Pass
Diazinon	0.10	0.20	<LOQ	Pass
Dichlorvos	0.50	1.00	<LOQ	Pass
Dimethoate	0.10	0.20	<LOQ	Pass
Ethoprophos	0.10	0.20	<LOQ	Pass
Etofenprox	0.20	0.40	<LOQ	Pass
Etoxazole	0.10	0.20	<LOQ	Pass
Fenoxycarb	0.10	0.20	<LOQ	Pass
Fenpyroximate	0.20	0.40	<LOQ	Pass
Fipronil	0.20	0.40	<LOQ	Pass
Flonicamid	0.50	1.00	<LOQ	Pass
Fludioxonil	0.20	0.40	<LOQ	Pass
Hexythiazox	0.50	1.00	<LOQ	Pass

Analyte	LOQ PPM	Limit PPM	Results PPM	Status
Imazalil	0.10	0.20	<LOQ	Pass
Imidacloprid	0.20	0.40	<LOQ	Pass
Kresoxim Methyl	0.20	0.40	<LOQ	Pass
Malathion	0.10	0.20	<LOQ	Pass
Metalaxyl	0.10	0.20	<LOQ	Pass
Methiocarb	0.10	0.20	<LOQ	Pass
Methomyl	0.20	0.40	<LOQ	Pass
Methyl Parathion	0.10	0.20	<LOQ	Pass
MGK-264	0.10	0.20	<LOQ	Pass
Myclobutanil	0.10	0.20	<LOQ	Pass
Naled	0.25	0.50	<LOQ	Pass
Oxamyl	0.50	1.00	<LOQ	Pass
Paclobutrazol	0.20	0.40	<LOQ	Pass
Permethrins	0.10	0.20	<LOQ	Pass
Phosmet	0.10	0.20	<LOQ	Pass
Piperonyl Butoxide	1.00	2.00	<LOQ	Pass
Prallethrin	0.10	0.20	<LOQ	Pass
Propiconazole	0.20	0.40	<LOQ	Pass
Propoxur	0.10	0.20	<LOQ	Pass
Pyrethrins	0.50	1.00	<LOQ	Pass
Pyridaben	0.10	0.20	<LOQ	Pass
Spinosad	0.10	0.20	<LOQ	Pass
Spiromesifen	0.10	0.20	<LOQ	Pass
Spirotetramat	0.10	0.20	<LOQ	Pass
Spiroxamine	0.20	0.40	<LOQ	Pass
Tebuconazole	0.20	0.40	<LOQ	Pass
Thiacloprid	0.10	0.20	<LOQ	Pass
Thiamethoxam	0.10	0.20	<LOQ	Pass
Trifloxystrobin	0.10	0.20	<LOQ	Pass

QC Batch ID: Pest-2020-10-02-01
Instrument: LC-MS/MS; Method: AOAC 2007.1-Mod; LOQ = Limit of Quantitation; The reported result is based on wet sample weight for that sample; Unless otherwise stated all quality control samples performed within specifications established by the Laboratory.Pesticide Analysis (Oregon Compliance Standard OAR 333-008-1190).

Ben Armstrong
QA Review

Confident Cannabis
All Rights Reserved
support@confidentcannabis.com
(866) 506-5866
www.confidentcannabis.com

Certificate of Analysis

Juniper Analytics LLC
1334 NE 2nd St
Bend, OR
(541) 382-3796
http://www.juniperanalyticsllc.com
ORELAP: License # 4101-001

Coastal Labs
, OR 97701
(619) 665-9966
Lic. #

Sample: 20JA1726.02

Concentrate/Extract Non Compliance
Residual Solvents-SOL-A, Pesticides-PES-A
and Potency-CAN

Report Date: 10/7/2020
Intake Date: 10/02/2020
Harvest/Prod. Date:
Solvents Analysis Date: 10/05/2020

CBG Isolate

Strain: CBG Isolate
METRC Sample: ; METRC Batch #: ;
External Batch #:CBGI006; Analytical Batch #: RS-2020-10-01-03

Residual Solvents

Pass

Analyte	LOQ	Limit	Results	Status
	PPM	PPM	PPM	
1,4-Dioxane	100	380.00	<LOQ	Pass
2-Butanol	500	5000.00	<LOQ	Pass
2-Ethoxy-Ethanol	100	160.00	<LOQ	Pass
2-Propanol IPA	500	5000.00	<LOQ	Pass
Acetone	500	5000.00	<LOQ	Pass
Acetonitrile	100	410.00	<LOQ	Pass
Benzene	1	2.00	<LOQ	Pass
Cumene	50	70.00	<LOQ	Pass
Cyclohexane	500	3880.00	<LOQ	Pass
Dichloromethane	100	600.00	<LOQ	Pass
Ethyl-Acetate	500	5000.00	<LOQ	Pass
Ethyl-Ether	500	5000.00	<LOQ	Pass
Ethylene Glycol	300	620.00	<LOQ	Pass
Ethylene Oxide	20	50.00	<LOQ	Pass
Heptane	500	5000.00	<LOQ	Pass
Isopropyl-Acetate	500	5000.00	<LOQ	Pass
Methanol	500	3000.00	<LOQ	Pass
Propane	500	5000.00	<LOQ	Pass
Tetrahydrofuran	100	720.00	<LOQ	Pass

Analyte	LOQ	Limit	Results	Status
	PPM	PPM	PPM	
Toluene	100	890.00	<LOQ	Pass
**Pentanes**	**500**	**5000.00**	**<LOQ**	**Pass**
n-Pentane			<LOQ	Tested
Isopentane			<LOQ	Tested
Neopentane			<LOQ	Tested
**Butanes**	**500**	**5000.00**	**<LOQ**	**Pass**
n-Butane			<LOQ	Tested
Isobutane			<LOQ	Tested
**Hexanes**	**50**	**290.00**	**<LOQ**	**Pass**
n-Hexane			<LOQ	Tested
2-Methyl-Pentane			<LOQ	Tested
3-Methyl-Pentane			<LOQ	Tested
2,2-Dimethyl-Butane			<LOQ	Tested
2,3-Dimethyl-Butane			<LOQ	Tested
**Xylenes**	**300**	**2170.00**	**<LOQ**	**Pass**
m-Xylene			<LOQ	Tested
o-Xylene			<LOQ	Tested
p-Xylene			<LOQ	Tested
Ethyl-Benzene			<LOQ	Tested

Tentatively Identified Compounds: None Detected

QC Batch ID: RS-2020-10-01-03
Instrument: GC/MS; Method: USP 467 - Modified; LOQ = Limit of Quantitation; The reported result is based on wet sample weight for that sample; Unless otherwise stated all quality control samples performed within specifications established by the Laboratory. Residual Solvent Analysis (Oregon Compliance Standard OAR 333-007-0410).

Ben Armstrong
QA Review

Confident Cannabis
All Rights Reserved
support@confidentcannabis.com
(866) 506-5866
www.confidentcannabis.com
