# Supplementary material for: Differential Effects of Cannabidiol and Cannabigerol on Cognition, Neuroinflammation, and Blood–Brain Barrier Integrity in a Rat Model of Iron Overload
Source: Neurotox Res. 2026 Aug 28;44(5):48. doi: 10.1007/s12640-026-00826-x (PMC13524965; doi:10.1007/s12640-026-00826-x)
Supplement: Supplementary file 2 [file 12640_2026_826_MOESM2_ESM.pdf]

CERTIFICATE OF ANALYSIS
ISO/IEC 17025:2017 ACCREDITATION #103104

Order #: 51914
Order Name: Purmed 4500mg
CBD Oil Tincture
Batch#: USP20445001
Received: 04/03/2020
Completed: 04/21/2020

PurMed Global LLC
95 NE 4th Avenue
Delray Beach FL, 33483
(888) 204-9353
www.purmedglobal.com

Sample

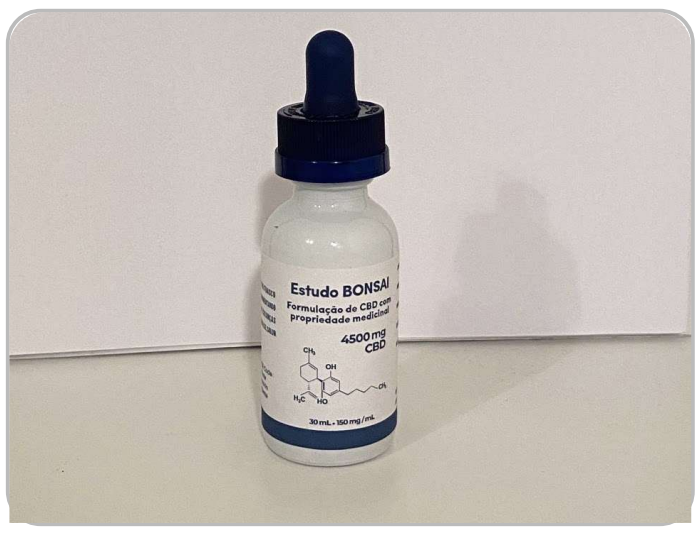

N/D	16.672%
D9-THC	Total CBD
**4,738.9 mg** Cannabinoids per bottle	**4,726.5 mg** CBD per bottle

1 bottle = 30 ml per bottle x density (0.945)
x Cannabinoid concentration

Cannabinoids Test

SHIMADZU INTEGRATED UPLC-PDA
GSL SOP 400 **PREPARED:** 04/03/2020 14:07:59

Cannabinoids	LOQ	weight(%)	mg/g	mg/bottle
D9-THC	10 PPM	N/D	N/D	N/D
THCA	10 PPM	N/D	N/D	N/D
CBD	10 PPM	**16.672%**	**166.721**	**4,726.5**
CBDA	20 PPM	N/D	N/D	N/D
CBDV	20 PPM	**0.044%**	**0.436**	**12.4**
CBC	10 PPM	N/D	N/D	N/D
CBN	10 PPM	N/D	N/D	N/D
CBG	10 PPM	N/D	N/D	N/D
CBGA	20 PPM	N/D	N/D	N/D
D8-THC	10 PPM	N/D	N/D	N/D
THCV	10 PPM	N/D	N/D	N/D
TOTAL D9-THC		**N/D**	**N/D**	**N/D**
TOTAL CBD*		**16.672%**	**166.721**	**4,726.5**
TOTAL CANNABINOIDS		**16.716%**	**167.157**	**4,738.9**

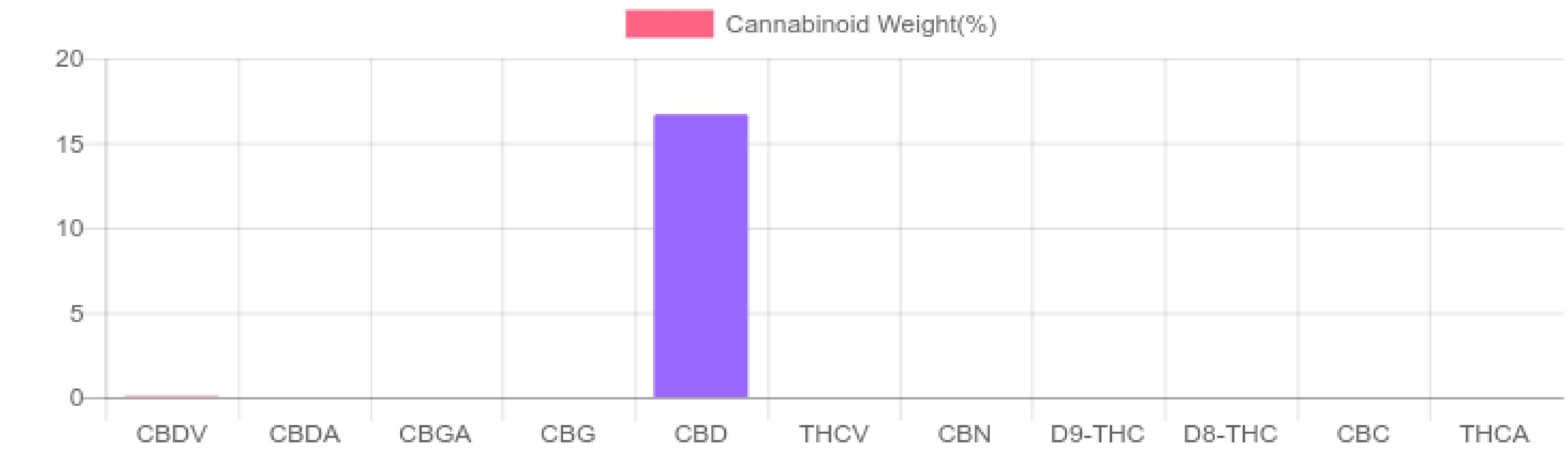

Reporting Limit 10 ppm
*Total CBD = CBD + CBDA x 0.877
N/D - Not Detected, B/LOQ - Below Limit of Quantification

Dr. Andrew Hall, Ph.D.,Chief Scientific Officer

Ben Witten, MS, MT., Lab Director

Green Scientific Labs
info@greenscientificlabs.com
1-833 TEST CBD

Green Scientific Labs uses its best efforts to deliver high quality results and to verify that the data contained therein are based on sound scientific judgment and levels listed are guidelines only and all data was reported based on standard laboratory procedures and deviations. However Green Scientific Labs makes no warranties or claims to that effect and further shall not be liable for any damage or misrepresentation that may result from the use or misuse of the data contained herein in any way. Further, Green Scientific Labs makes no claims regarding representations of the analyzed sample to the larger batch from which it was taken. Data and information in this report are intended solely for the individual(s) for whom samples were submitted and as part of our strict confidentiality policy, Green Scientific Labs can only discuss results with the original client of record.

CERTIFICATE OF ANALYSIS
ISO/IEC 17025:2017 ACCREDITATION #103104

Order #: 51914
Order Name: Purmed 4500mg CBD Oil Tincture
Batch#: USP20445001
Received: 04/03/2020
Completed: 04/21/2020

PurMed Global LLC
95 NE 4th Avenue
Delray Beach FL, 33483
(888) 204-9353
www.purmedglobal.com

PESTICIDE ANALYSIS:

GSL SOP 401 **PREPARED:** 04/03/2020 13:21:00

GCMS-MS - Shimadzu GCMS-TQ8040

Pesticide	Action Level (ppm)	Results (ppm)	LOQ (ppm)	LOD (ppm)
CAPTAN	0.700	N/D	0.003	0.001
CHLORDANE	0.100	N/D	0.003	0.001
CHLORFENAPYR	0.100	N/D	0.003	0.001
COUMAPHOS	0.100	N/D	0.003	0.001
CYFLUTHRIN	2.000	N/D	0.003	0.001
CYPERMETHRIN	1.000	N/D	0.003	0.001
PARATHION-METHYL	0.100	N/D	0.003	0.001
PENTACHLORONITROBENZENE	0.100	N/D	0.003	0.001

LCMS-MS - Shimadzu LCMS-8060

Pesticide	Action Level (ppm)	Results (ppm)	LOQ (ppm)	LOD (ppm)
ABAMECTIN B1A	0.100	N/D	0.005	0.001
ACEPHATE	0.100	N/D	0.001	0.001
ACEQUINOCYL	0.100	N/D	0.001	0.001
ACETAMIPRID	0.100	N/D	0.005	0.001
ALDICARB	0.100	N/D	0.005	0.001
AZOXYSTROBIN	0.100	N/D	0.001	0.001
BIFENAZATE	0.100	N/D	0.005	0.001
BIFENTHRIN	3.000	N/D	0.005	0.001
BOSCALID	0.100	N/D	0.005	0.001
CARBARYL	0.500	N/D	0.003	0.001
CARBOFURAN	0.100	N/D	0.001	0.001
CHLORANTRANILIPROLE	10.000	N/D	0.005	0.005
CHLORPYRIFOS	0.100	N/D	0.001	0.001
CLOFENTEZINE	0.100	N/D	0.001	0.001
DAMINOZIDE	0.100	N/D	0.005	0.001
DIAZINON	0.100	N/D	0.001	0.001
DICHLORVOS	0.100	N/D	0.005	0.001
DIMETHOATE	0.100	N/D	0.001	0.001
DIMETHOMORPH	2.000	N/D	0.005	0.001
ETHOPROPHOS	0.100	N/D	0.001	0.001
ETOFENPROX	0.100	N/D	0.001	0.001
ETOXAZOLE	0.100	N/D	0.010	0.005
FENHEXAMID	0.100	N/D	0.005	0.001
FENOXYCARB	0.100	N/D	0.005	0.001
FENPYROXIMATE	0.100	N/D	0.001	0.001
FIPRONIL	0.100	N/D	0.003	0.001
FLONICAMID	0.100	N/D	0.025	0.010
FLUDIOXONIL	0.100	N/D	0.003	0.001
HEXYTHIAZOX	0.100	N/D	0.005	0.001
IMAZALIL	0.100	N/D	0.005	0.001
IMIDACLOPRID	5.000	N/D	0.005	0.001
KRESOXIM-METHYL	0.100	N/D	0.010	0.005
MALATHION	0.500	N/D	0.005	0.001
METALAXYL	2.000	N/D	0.001	0.001
METHIOCARB	0.100	N/D	0.005	0.001
METHOMYL	1.000	N/D	0.001	0.001
MEVINPHOS	0.100	N/D	0.001	0.001
MYCLOBUTANIL	0.100	N/D	0.005	0.001
NALED	0.100	N/D	0.005	0.001
OXAMYL	0.500	N/D	0.001	0.001
PACLOBUTRAZOL	0.100	N/D	0.005	0.001
PERMETHRINS	0.500	N/D	0.005	0.001
PHOSMET	0.100	N/D	0.005	0.001
PIPERONYL BUTOXIDE	3.000	N/D	0.001	0.001
PRALLETHRIN	0.100	N/D	0.005	0.005
PROPICONAZOLE	0.100	N/D	0.010	0.005
PROPOXUR	0.100	N/D	0.001	0.001
PYRETHRINS (PYRETHRIN I)	0.500	N/D	0.005	0.005
PYRIDABEN	0.100	N/D	0.005	0.001
SPINETORAM	0.100	N/D	0.001	0.001
SPINOSAD	0.100	N/D	0.001	0.001
SPIROMESIFEN	0.100	N/D	0.005	0.001
SPIROTETRAMAT	0.100	N/D	0.001	0.001
SPIROXAMINE	0.100	N/D	0.001	0.001
TEBUCONAZOLE	0.100	N/D	0.005	0.001
THIACLOPRID	0.100	N/D	0.001	0.001
THIAMETHOXAM	5.000	N/D	0.001	0.001
TRIFLOXYSTROBIN	0.100	N/D	0.001	0.001

N/D = Not Detected, A/LOQ = Above LOQ Level, B/LOQ = Below LOQ Level, B/LOD = Below LOD Level

Dr. Andrew Hall, Ph.D.,Chief Scientific Officer

Ben Witten, MS, MT., Lab Director

Green Scientific Labs
info@greenscientificlabs.com
1-833 TEST CBD

Green Scientific Labs uses its best efforts to deliver high quality results and to verify that the data contained therein are based on sound scientific judgment and levels listed are guidelines only and all data was reported based on standard laboratory procedures and deviations. However Green Scientific Labs makes no warranties or claims to that effect and further shall not be liable for any damage or misrepresentation that may result from the use or misuse of the data contained herein in any way. Further, Green Scientific Labs makes no claims regarding representations of the analyzed sample to the larger batch from which it was taken. Data and information in this report are intended solely for the individual(s) for whom samples were submitted and as part of our strict confidentiality policy, Green Scientific Labs can only discuss results with the original client of record.

CERTIFICATE OF ANALYSIS
ISO/IEC 17025:2017 ACCREDITATION #103104

Order #: 51914
Order Name: Purmed 4500mg CBD Oil Tincture
Batch#: USP20445001
Received: 04/03/2020
Completed: 04/21/2020

PurMed Global LLC
95 NE 4th Avenue
Delray Beach FL, 33483
(888) 204-9353
www.purmedglobal.com

RESIDUAL SOLVENTS:

Headspace GCMS - Shimadzu GCMS QP2020 with HS20

GSL SOP 405
Prepared: 04/03/2020 18:08:54

Residual Solvent	Action Level (ppm)	Results (ppm)	LOQ (ppm)	LOD (ppm)
ACETONE	5,000	N/D	140	20
ACETONITRILE	410	N/D	25	1
BENZENE	1	N/D	1	0.5
BUTANE	5,000	N/D	50	10
CHLOROFORM	1	N/D	1	0.5
DICHLOROETHANE	1	N/D	1	0.5
DICHLOROMETHANE	1	N/D	1	0.5
ETHANOL	5,000	N/D	140	20
ETHYL ACETATE	5,000	N/D	140	20
ETHYL ETHER	5,000	N/D	140	20
ISOPROPYL ALCOHOL	5,000	N/D	140	20
METHANOL	3,000	N/D	100	20
N-HEPTANE	5,000	N/D	140	20
N-HEXANE	290	N/D	18	10
PENTANE	5,000	B/LOQ	140	20
PROPANE	5,000	N/D	20	1
TOLUENE	890	N/D	53	1
TRICHLOROETHENE	1	N/D	1	0.5
XYLENES	150	N/D	130	20

Dr. Andrew Hall, Ph.D.,Chief Scientific Officer

Ben Witten, MS, MT., Lab Director

Green Scientific Labs
info@greenscientificlabs.com
1-833 TEST CBD

Green Scientific Labs uses its best efforts to deliver high quality results and to verify that the data contained therein are based on sound scientific judgment and levels listed are guidelines only and all data was reported based on standard laboratory procedures and deviations. However Green Scientific Labs makes no warranties or claims to that effect and further shall not be liable for any damage or misrepresentation that may result from the use or misuse of the data contained herein in any way. Further, Green Scientific Labs makes no claims regarding representations of the analyzed sample to the larger batch from which it was taken. Data and information in this report are intended solely for the individual(s) for whom samples were submitted and as part of our strict confidentiality policy, Green Scientific Labs can only discuss results with the original client of record.

CERTIFICATE OF ANALYSIS
ISO/IEC 17025:2017 ACCREDITATION #103104

Order #: 51914
Order Name: Purmed 4500mg CBD Oil Tincture
Batch#: USP20445001
Received: 04/03/2020
Completed: 04/21/2020

PurMed Global LLC
95 NE 4th Avenue
Delray Beach FL, 33483
(888) 204-9353
www.purmedglobal.com

Microbial Analysis:

Microbial Analysis GSL SOP 406

Uploaded: 04/09/2020 09:13:20

PCR - Agilent AriaMX

Test	Test Method Used	Device Used	LOD	Allowable Criteria	Actual Result	Pass/Fail
STEC E.COLI*	USP 61/62†	ARIAMX PCR	2 COPIES OF DNA	PRESENCE / ABSENT	BELOW LOD	PASS
SALMONELLA*	USP 61/62†	ARIAMX PCR	5 COPIES OF DNA	PRESENCE / ABSENT	BELOW LOD	PASS
ASPERGILLUS	USP 61/62†	ARIAMX PCR	ASP_LOD***	PRESENCE / ABSENT	BELOW LOD	PASS
YEAST AND MOLD	USP 61/62†	ARIAMX PCR	363.05518 CFU/G**	1,000	BELOW THRESHOLD	PASS
TOTAL AEROBIC BACTERIA	USP 61/62†	ARIAMX PCR	0.25316 CFU/G**	10,000	BELOW THRESHOLD	PASS
COLIFORM	USP 61/62†	ARIAMX PCR	3.41539 CFU/G**	100	BELOW THRESHOLD	PASS
ENTEROBACTERIACEAE	USP 61/62†	ARIAMX PCR	0.32951 CFU/G**	100	BELOW THRESHOLD	PASS

† USP 61 (enumeration of bacteria TAC, TYM, and ENT/Coliform), USP 62 (identifying specific species E.coli Aspergillus etc)
* STEC and Salmonella run as Multiplex
** CFU/g Calculation based on Select Category Type Gummy MIP/Extract Flower matrix
*** Flavus = 2 Copies of DNA / Fumigatis = 2 Copies of DNA Niger = 20 Copies of DNA / Terrus = 10 copies of DNA

Dr. Andrew Hall, Ph.D.,Chief Scientific Officer

Ben Witten, MS, MT., Lab Director

Green Scientific Labs
info@greenscientificlabs.com
1-833 TEST CBD

PJLA Testing Accreditation #103104

Green Scientific Labs uses its best efforts to deliver high quality results and to verify that the data contained therein are based on sound scientific judgment and levels listed are guidelines only and all data was reported based on standard laboratory procedures and deviations. However Green Scientific Labs makes no warranties or claims to that effect and further shall not be liable for any damage or misrepresentation that may result from the use or misuse of the data contained herein in any way. Further, Green Scientific Labs makes no claims regarding representations of the analyzed sample to the larger batch from which it was taken. Data and information in this report are intended solely for the individual(s) for whom samples were submitted and as part of our strict confidentiality policy, Green Scientific Labs can only discuss results with the original client of record.

CERTIFICATE OF ANALYSIS
ISO/IEC 17025:2017 ACCREDITATION #103104

Order #: 51914
Order Name: Purmed 4500mg CBD Oil Tincture
Batch#: USP20445001
Received: 04/03/2020
Completed: 04/21/2020

PurMed Global LLC
95 NE 4th Avenue
Delray Beach FL, 33483
(888) 204-9353
www.purmedglobal.com

Mycotoxin Analysis:

LC-MS - Shimadzu LCMS-8060
GSL SOP 401

Uploaded: 04/06/2020 10:26:51

Analyte	Action Lvl (ppb)	Results (ppb)
AFLATOXIN B1	20	N/D
AFLATOXIN B2	20	N/D
AFLATOXIN G1	20	N/D
AFLATOXIN G2	20	N/D
OCHRATOXIN A	20	N/D

LOQ is 4ppb, LOD is 1ppb

Heavy Metals Analysis:

ICP-MS - Shimadzu ICPMS-2030
GSL SOP 403

Uploaded: 04/06/2020 17:46:50

Metal	Action Level (ppb)	Result (ppb)
ARSENIC (AS)	200	B/LOQ
CADMIUM (CD)	200	B/LOQ
MERCURY (HG)	100	B/LOQ
LEAD (PB)	500	B/LOQ

Lower Limit of Quantitation (LOQ) is 75 ppb

Water Activity:

HD-3A Water Activity Meter - GSL SOP 407

Uploaded: 04/03/2020 18:14:32

Water
0.47

Filth and Foreign Materials Analysis:

Microscope - Amscope Simul-Focal Trinocular Stereo Zoom Microscope - GSL SOP 409

Uploaded: 04/03/2020 18:16:50

Pass: No Foreign Materials Detected

Dr. Andrew Hall, Ph.D.,Chief Scientific Officer

Ben Witten, MS, MT., Lab Director

Green Scientific Labs
info@greenscientificlabs.com
1-833 TEST CBD

PJLA Testing Accreditation #103104

Green Scientific Labs uses its best efforts to deliver high quality results and to verify that the data contained therein are based on sound scientific judgment and levels listed are guidelines only and all data was reported based on standard laboratory procedures and deviations. However Green Scientific Labs makes no warranties or claims to that effect and further shall not be liable for any damage or misrepresentation that may result from the use or misuse of the data contained herein in any way. Further, Green Scientific Labs makes no claims regarding representations of the analyzed sample to the larger batch from which it was taken. Data and information in this report are intended solely for the individual(s) for whom samples were submitted and as part of our strict confidentiality policy, Green Scientific Labs can only discuss results with the original client of record.
