# Supplementary material for: Differential Effects of Cannabidiol and Cannabigerol on Cognition, Neuroinflammation, and Blood–Brain Barrier Integrity in a Rat Model of Iron Overload
Source: Neurotox Res. 2026 Aug 28;44(5):48. doi: 10.1007/s12640-026-00826-x (PMC13524965; doi:10.1007/s12640-026-00826-x)

Supplementary Figure S3. Full-length western blot images of Occludin (~54 kDa) and GAPDH (37 kDa) in the hippocampus of adult rats subjected to neonatal treatment with sorbitol or iron, followed by adult treatment with vehicle (VEH), cannabidiol (CBD), or cannabigerol (CBG). GAPDH was used as the loading control and detected on the same membranes as Occludin. The images represent the membranes used for protein quantification. For each membrane, two images acquired at different exposure times are shown, corresponding to the exposure used for densitometric quantification of GAPDH and Occludin, respectively. Samples labeled "Not used for quantification" were excluded because they did not meet the criteria for reliable quantification or presented technical issues. Some samples were subsequently reanalyzed on another membrane and therefore appear in more than one gel.

GEL 1

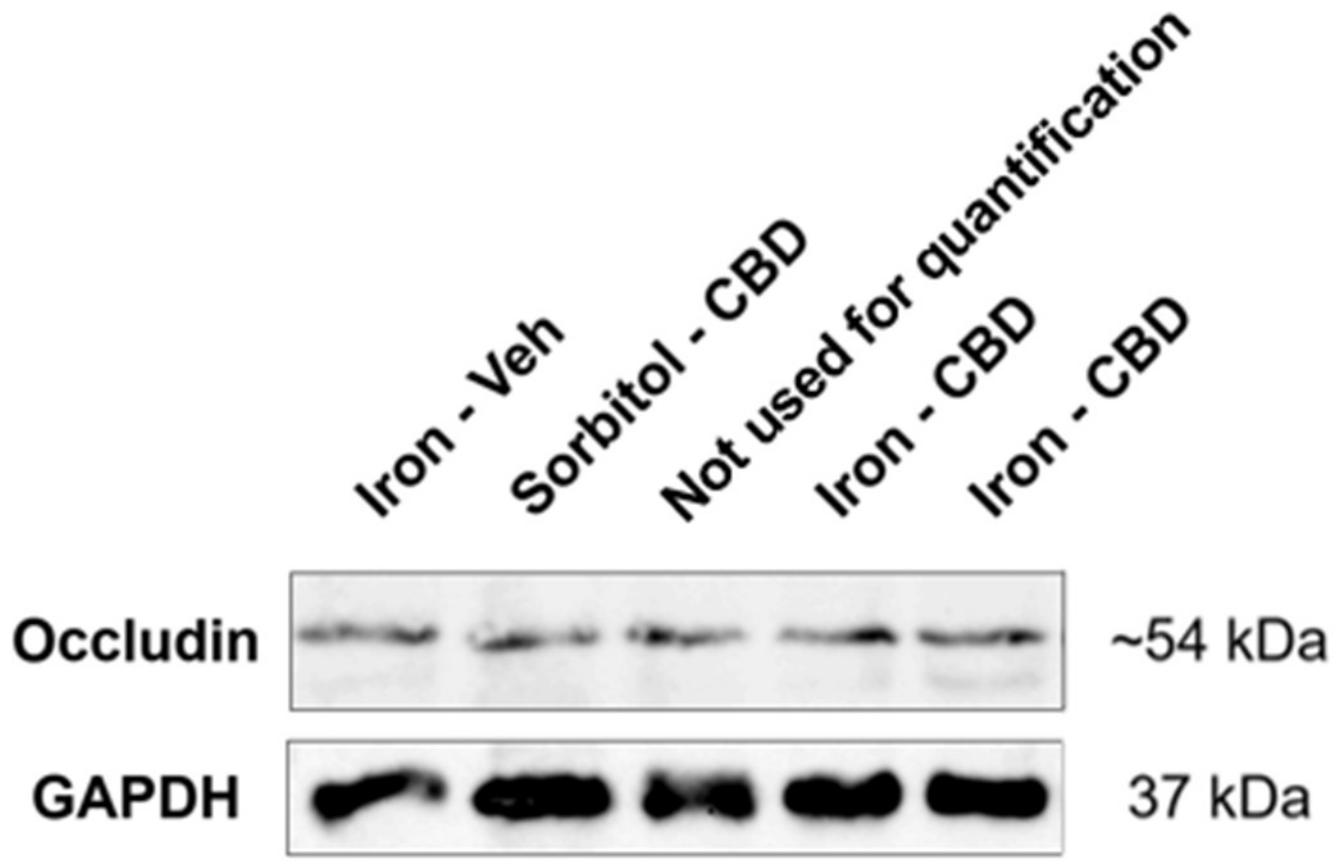

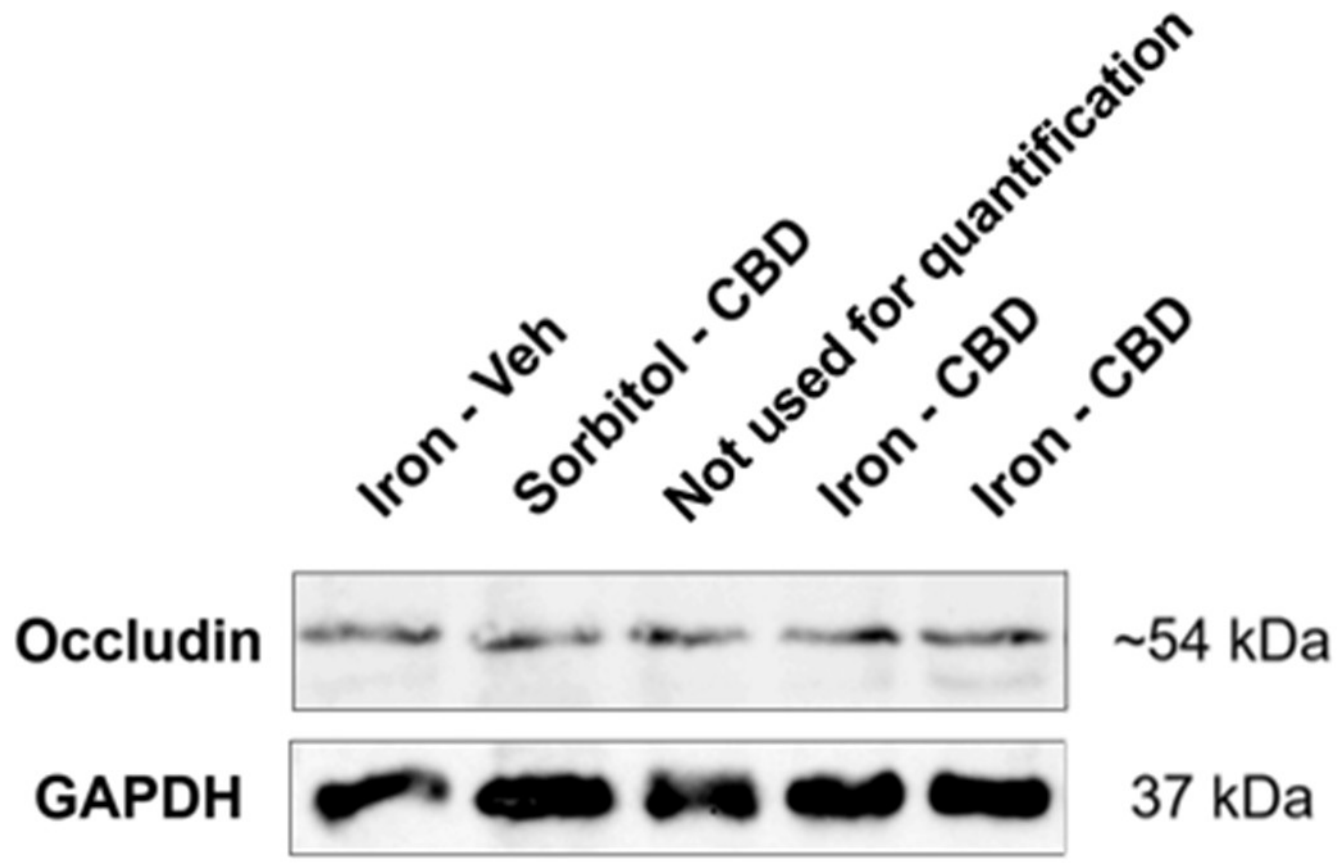

Exposure optimized for GAPDH

Exposure optimized for Occludin

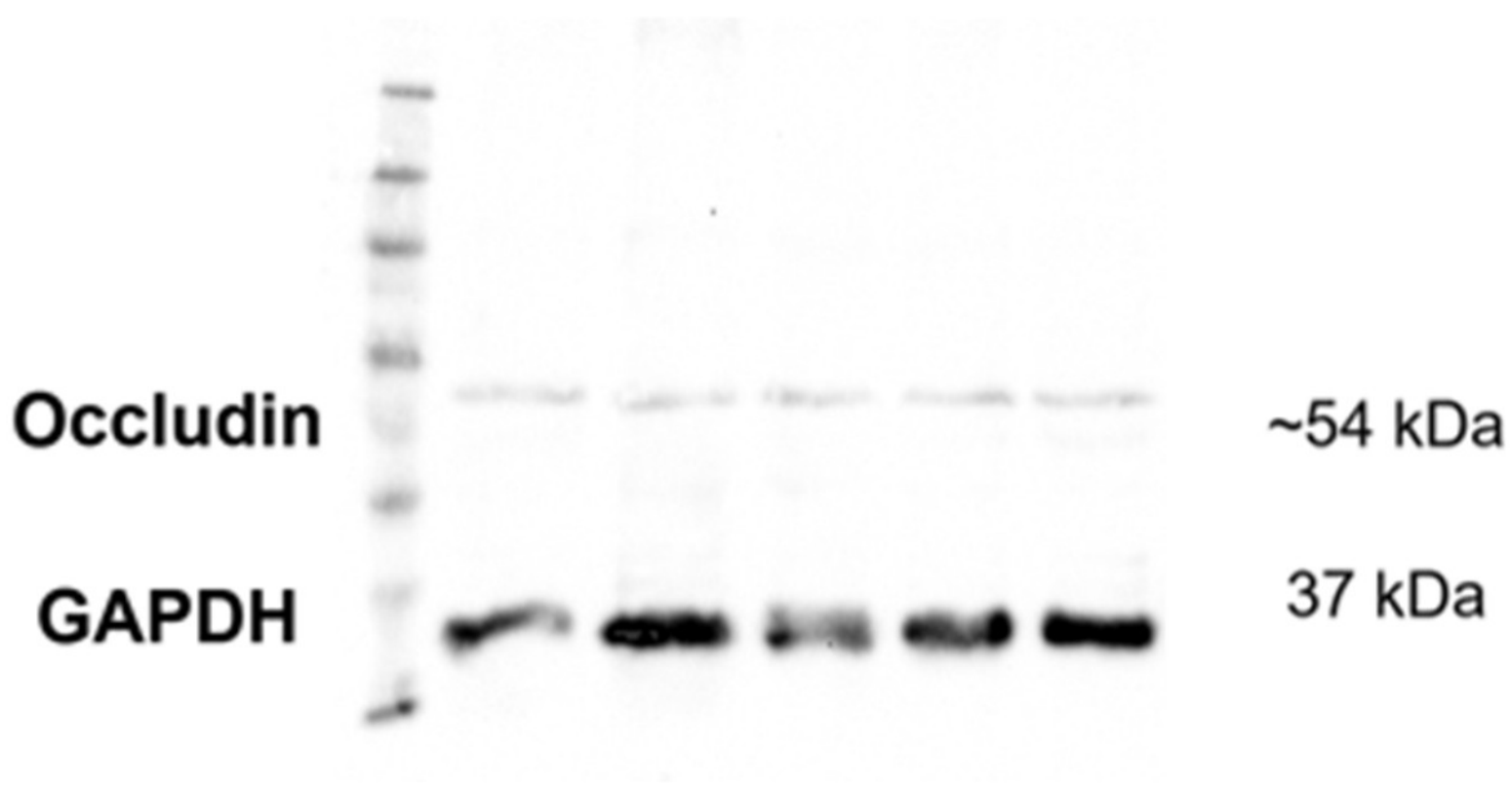

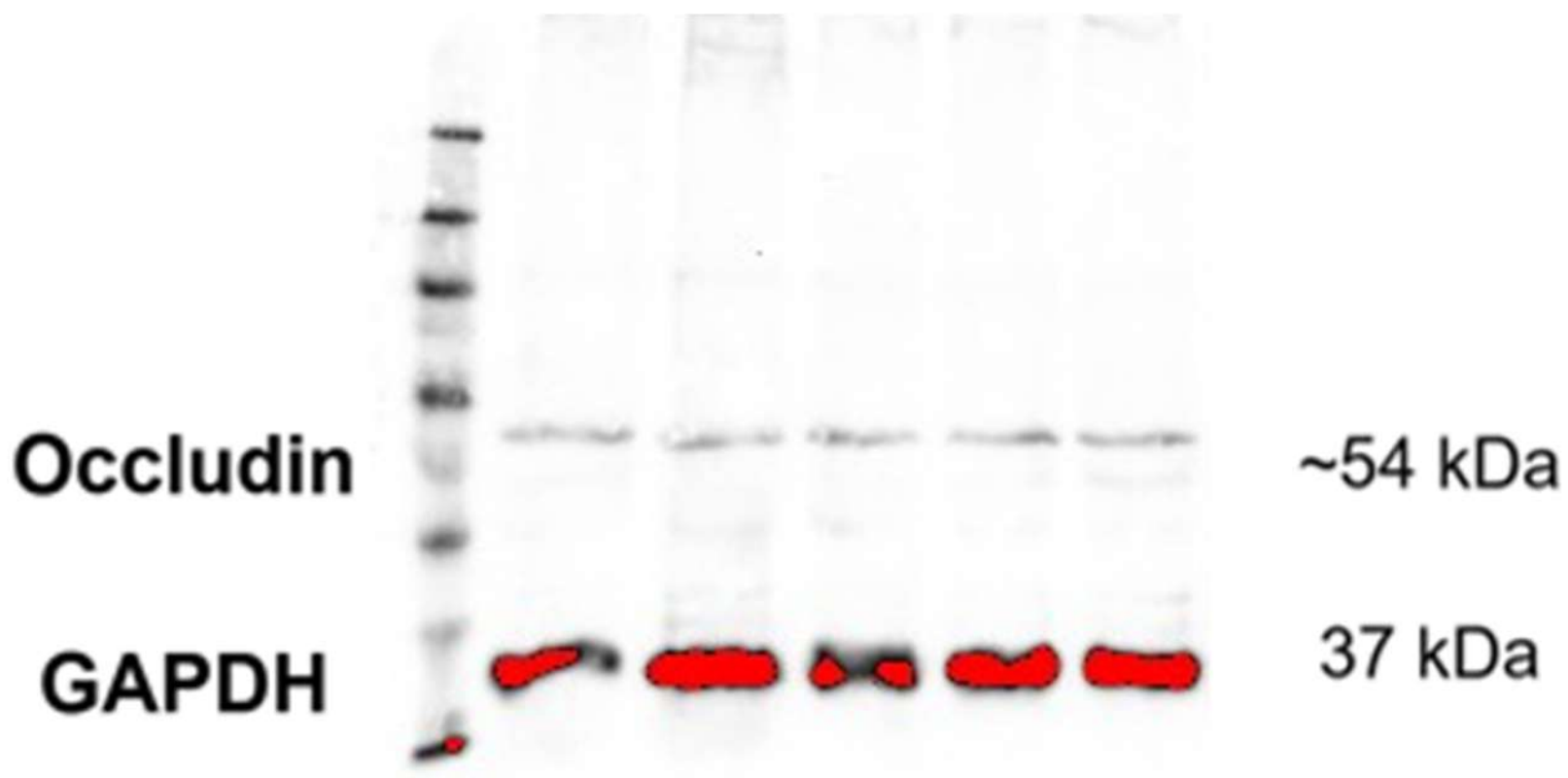

GEL 2

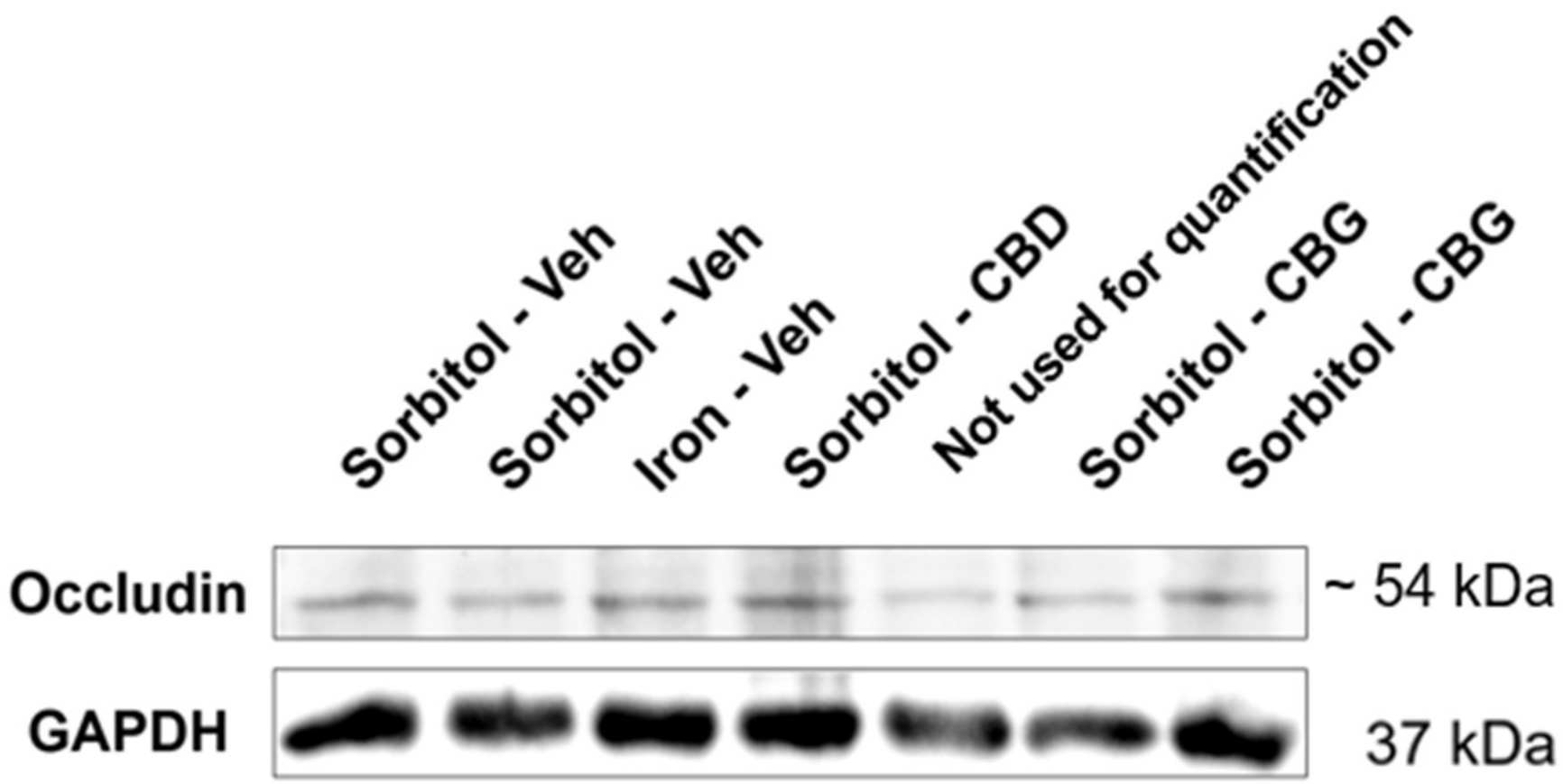

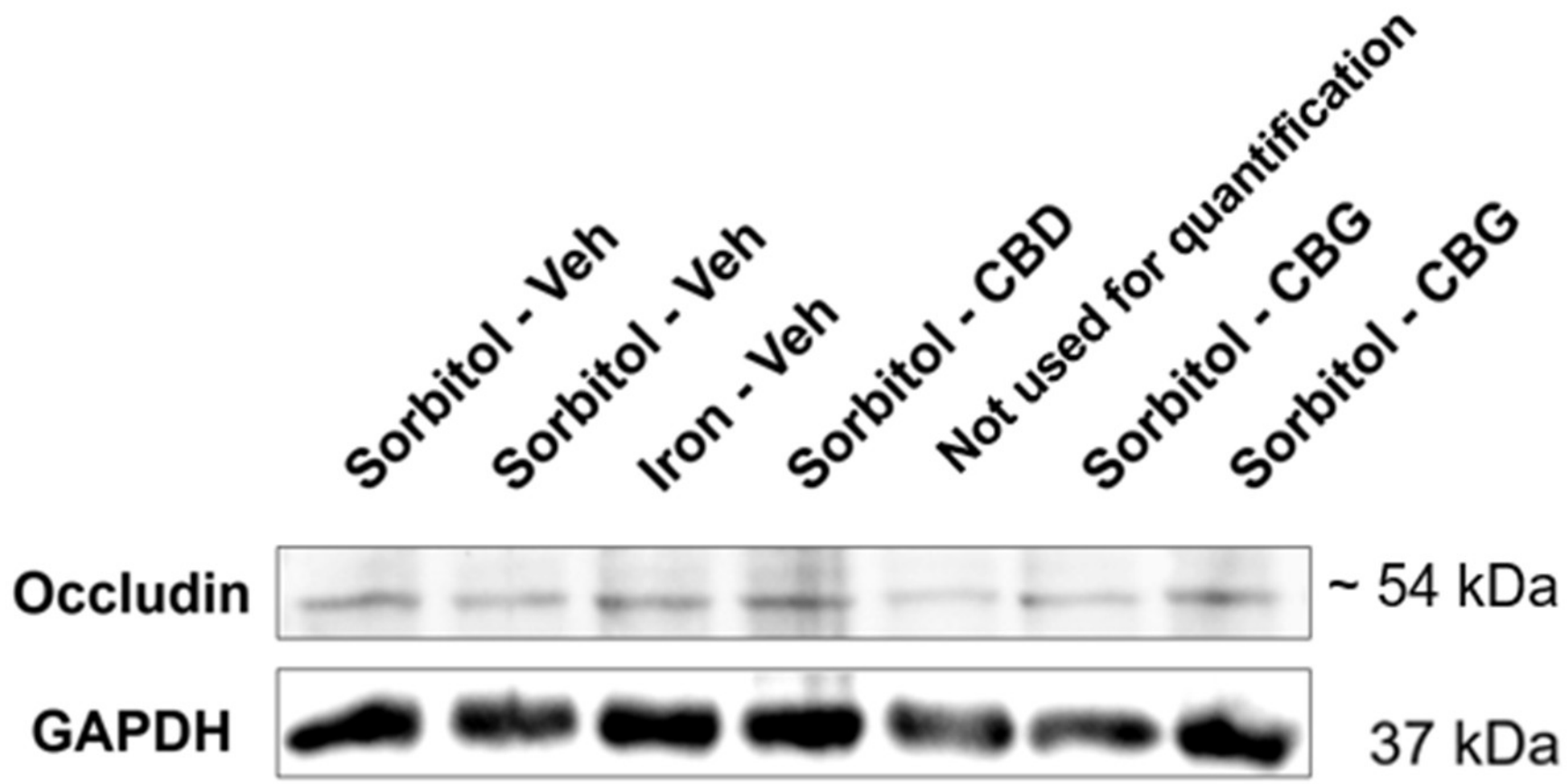

Exposure optimized for GAPDH

Exposure optimized for Occludin

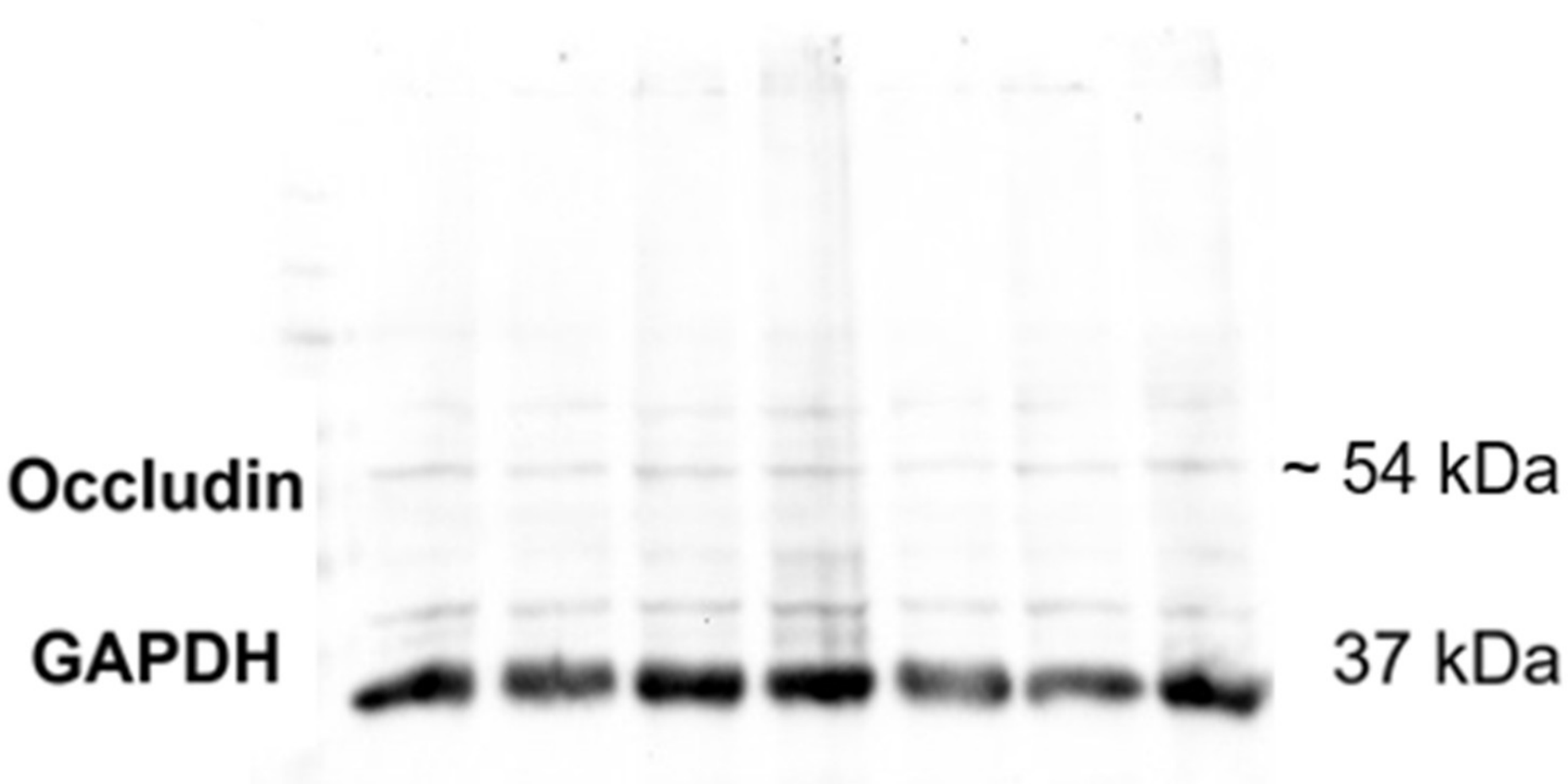

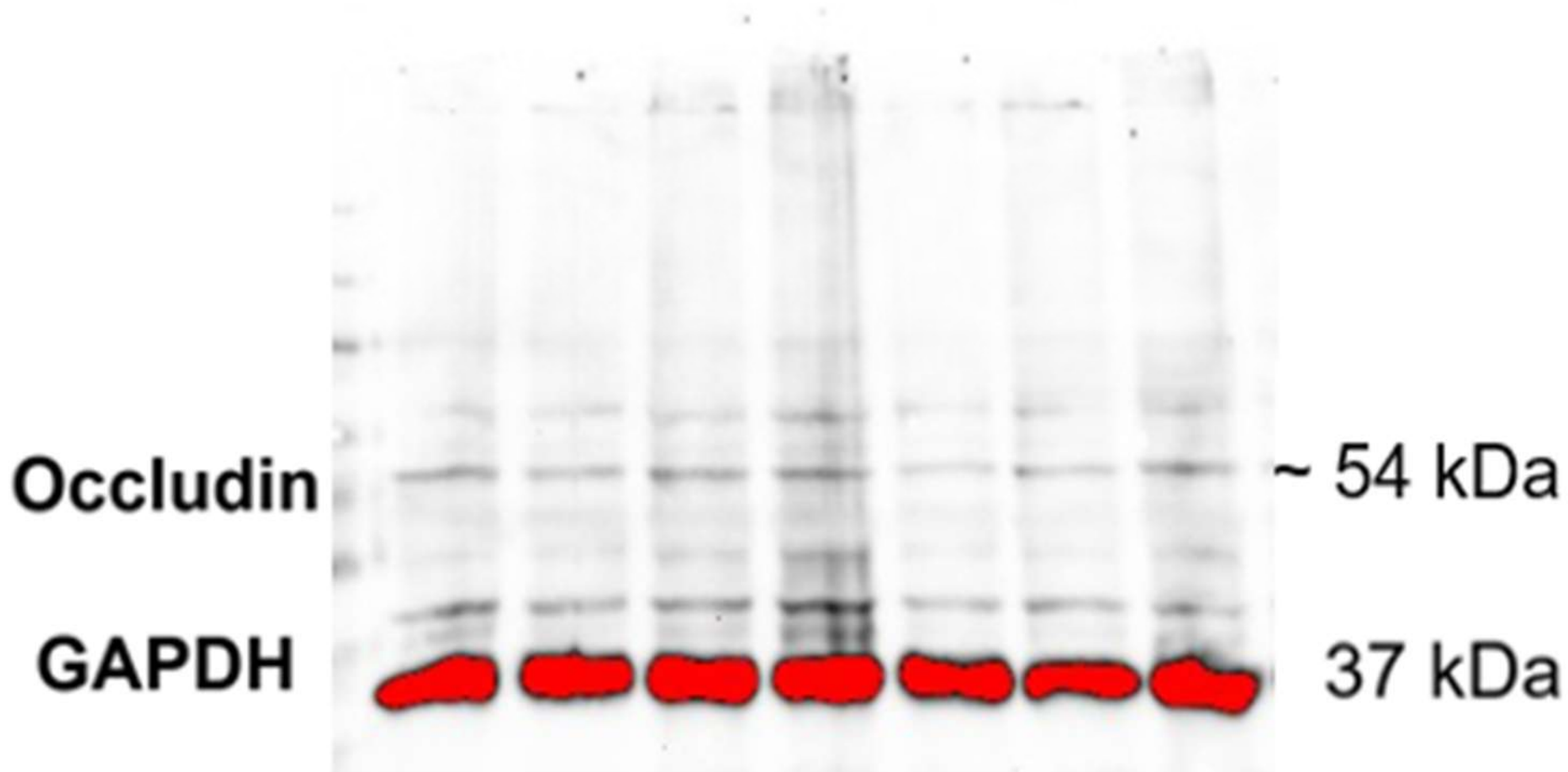

GEL 3

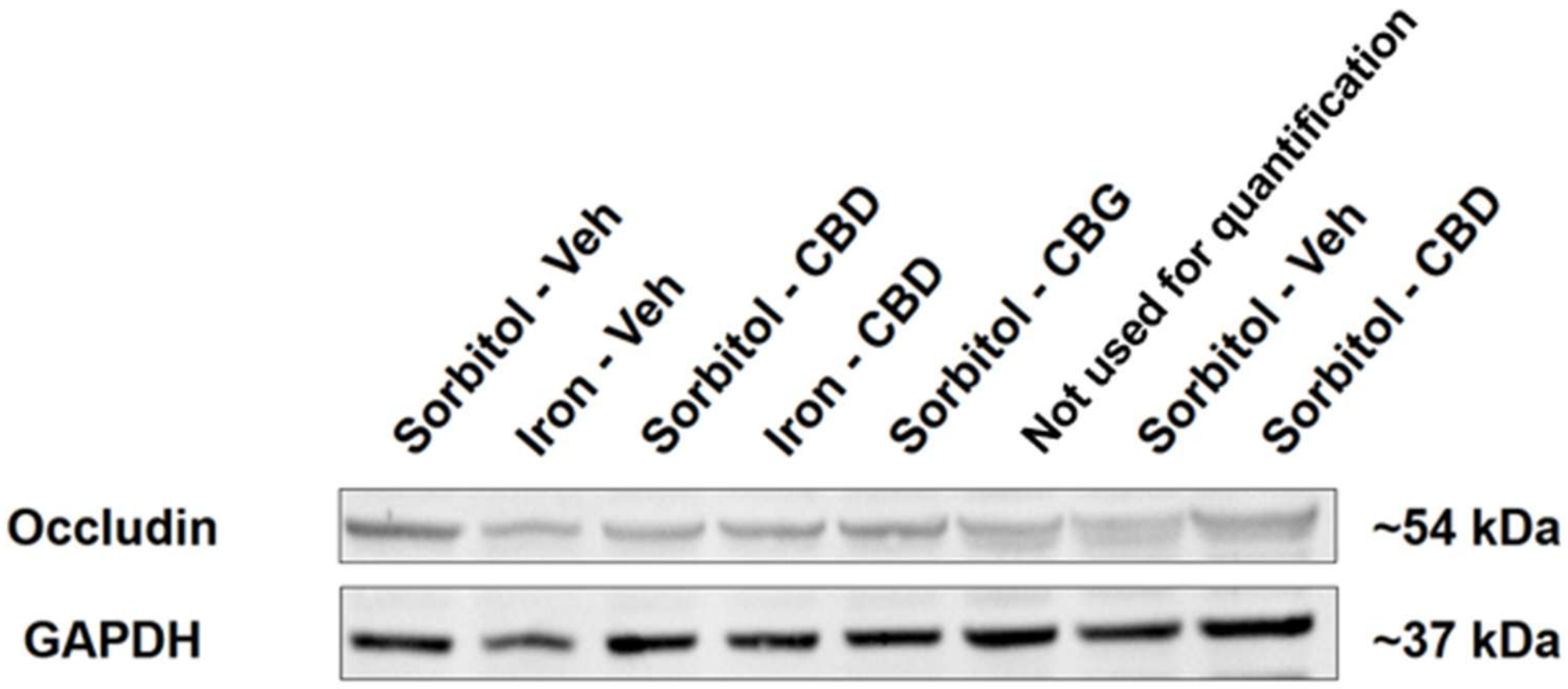

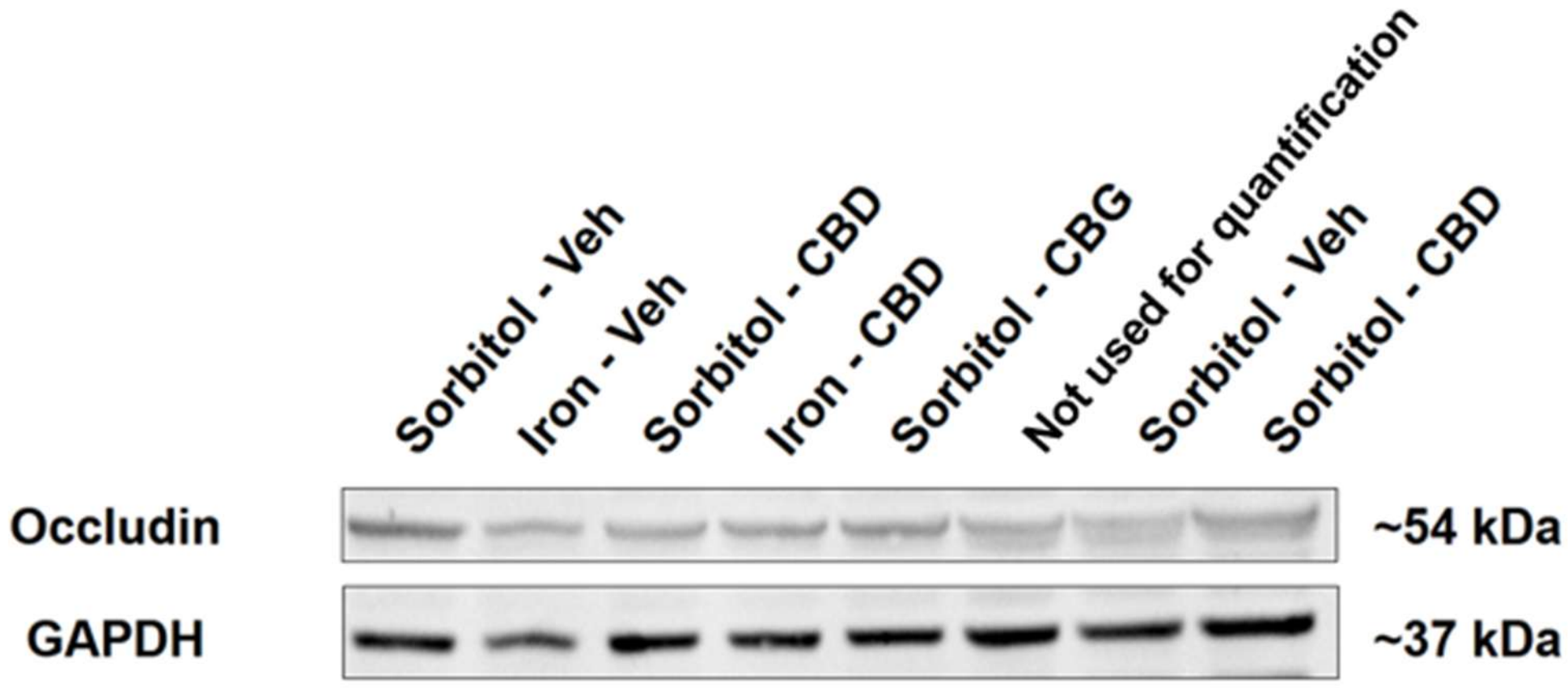

Exposure optimized for GAPDH

Exposure optimized for Occludin

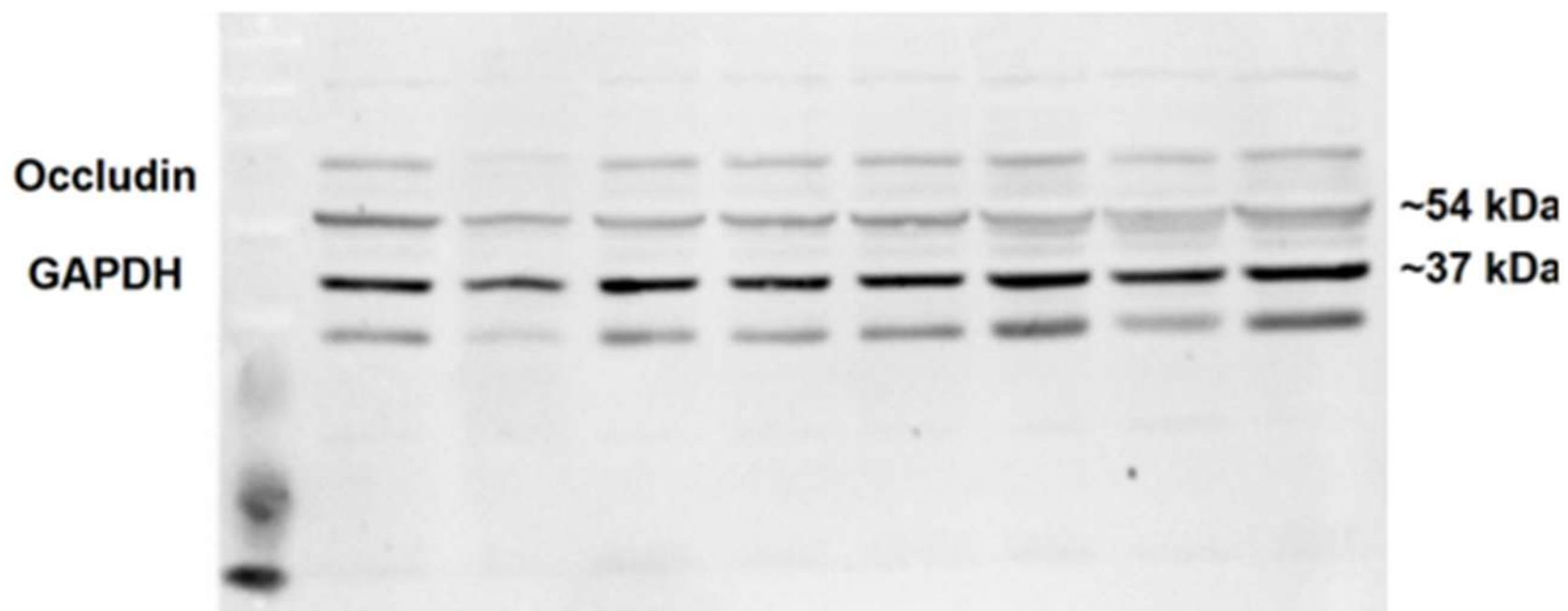

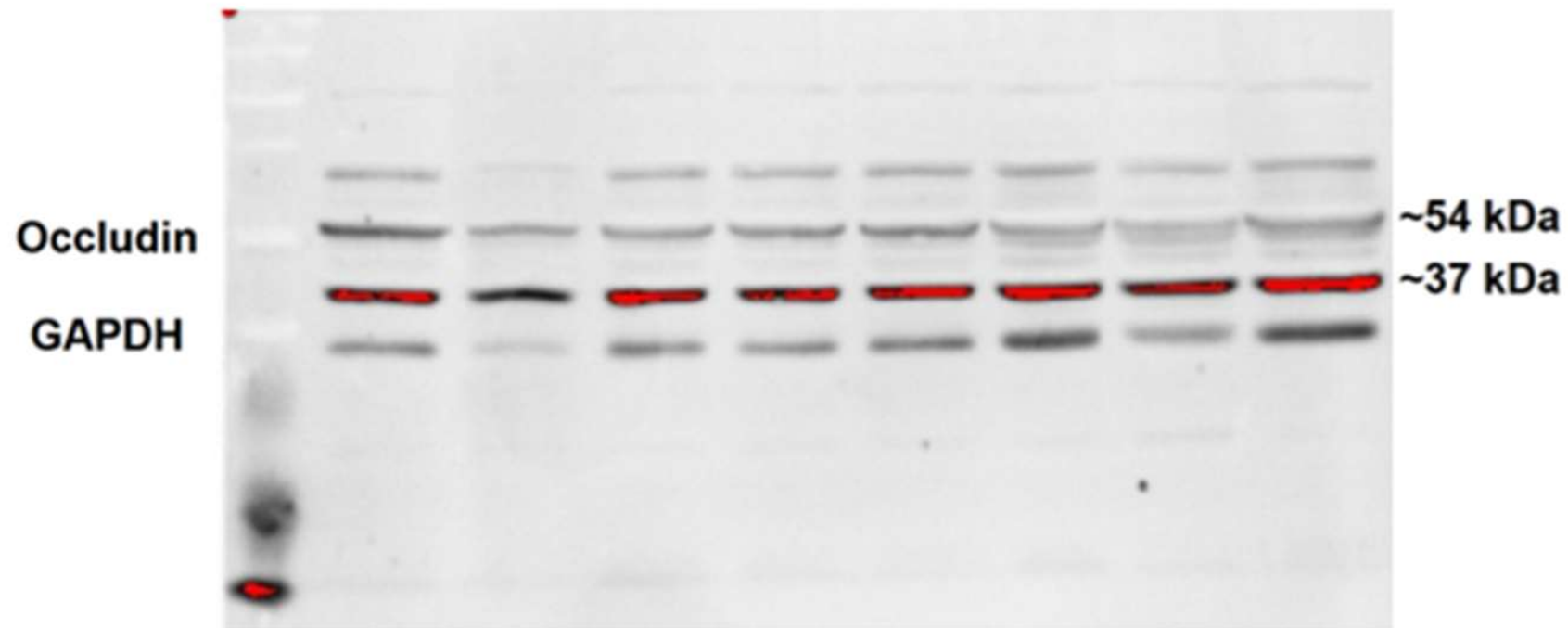

GEL 4

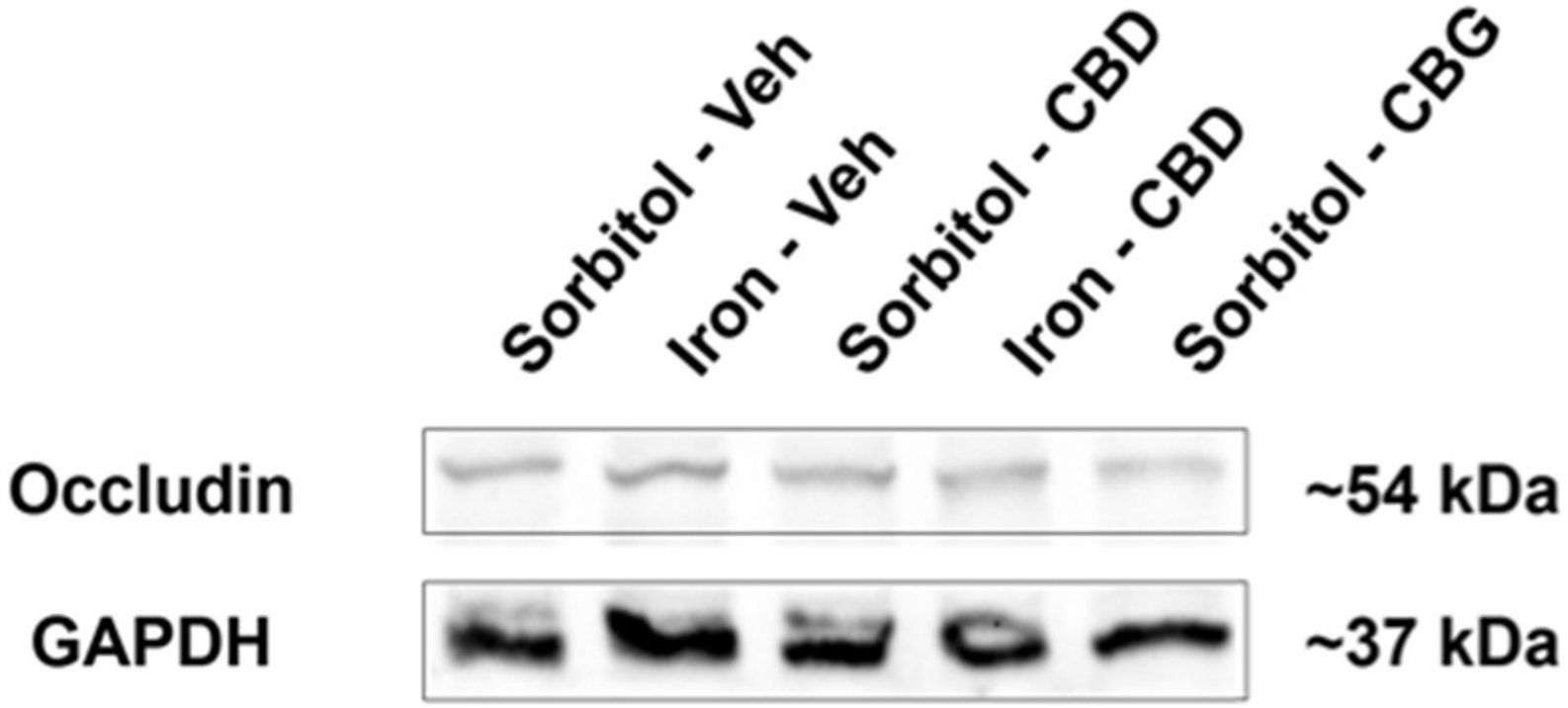

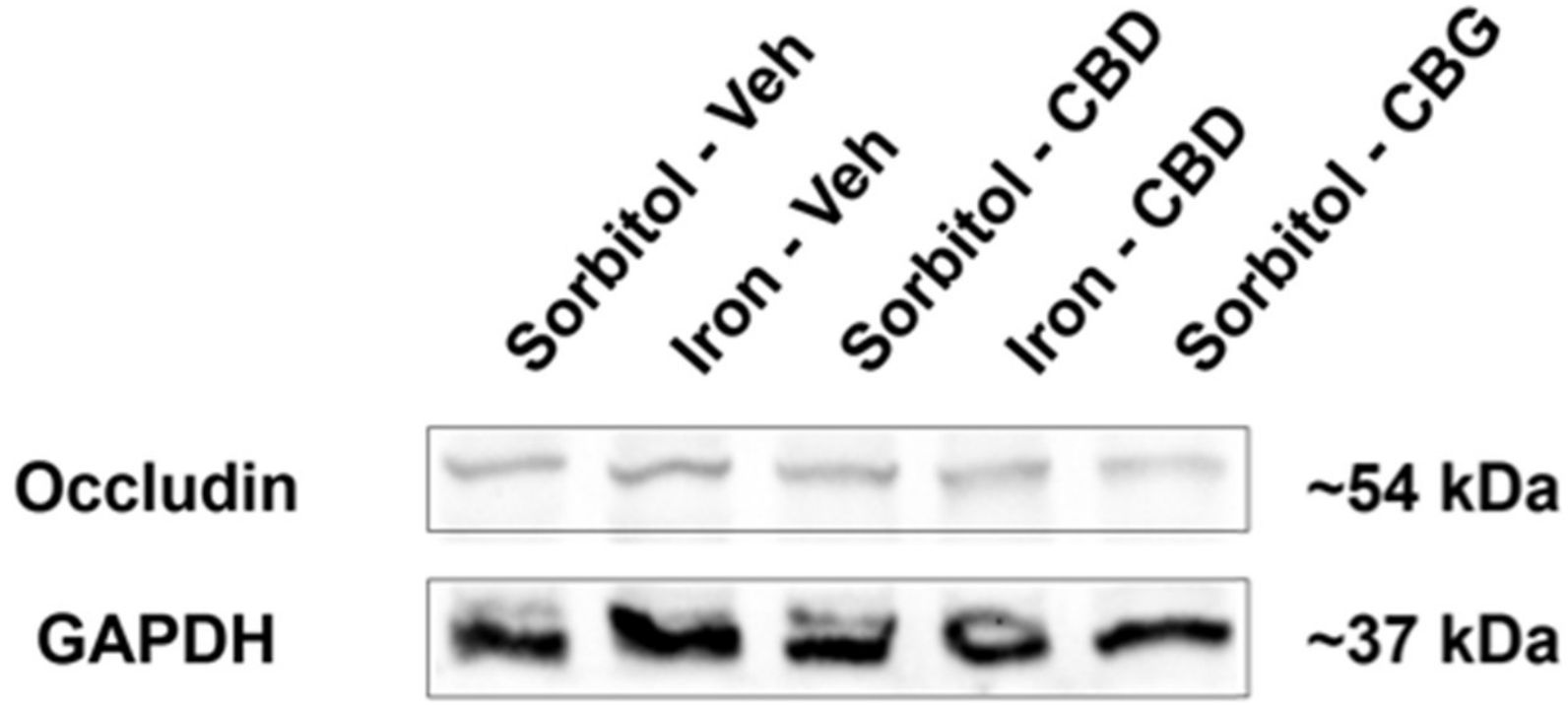

Exposure optimized for GAPDH

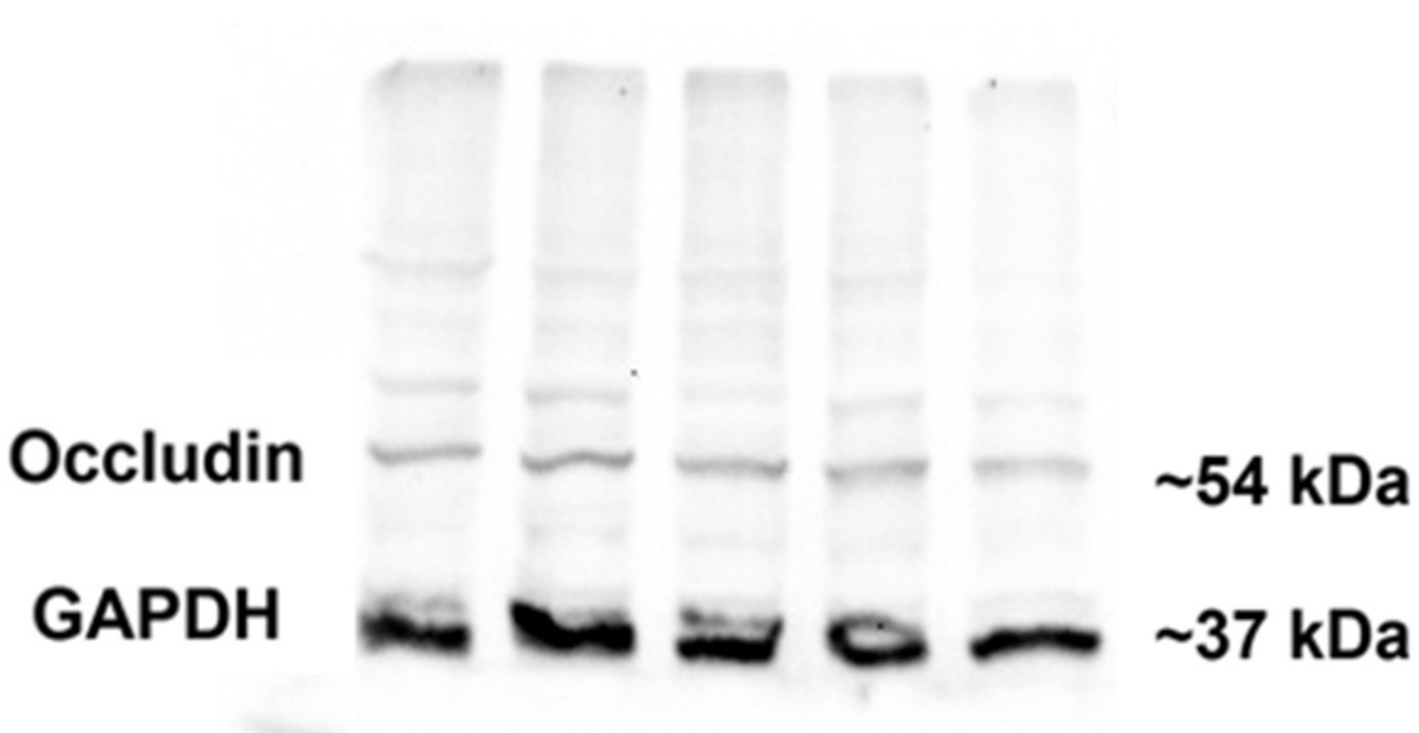

Exposure optimized for Occludin

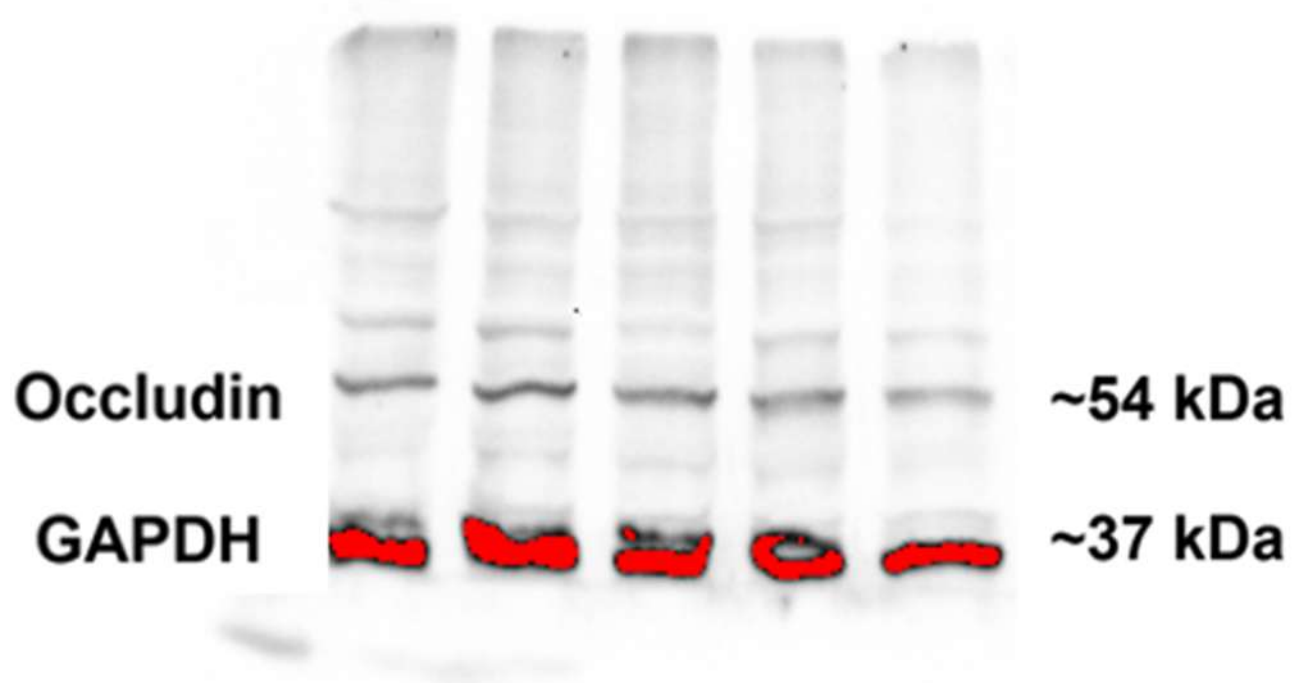

GEL 5

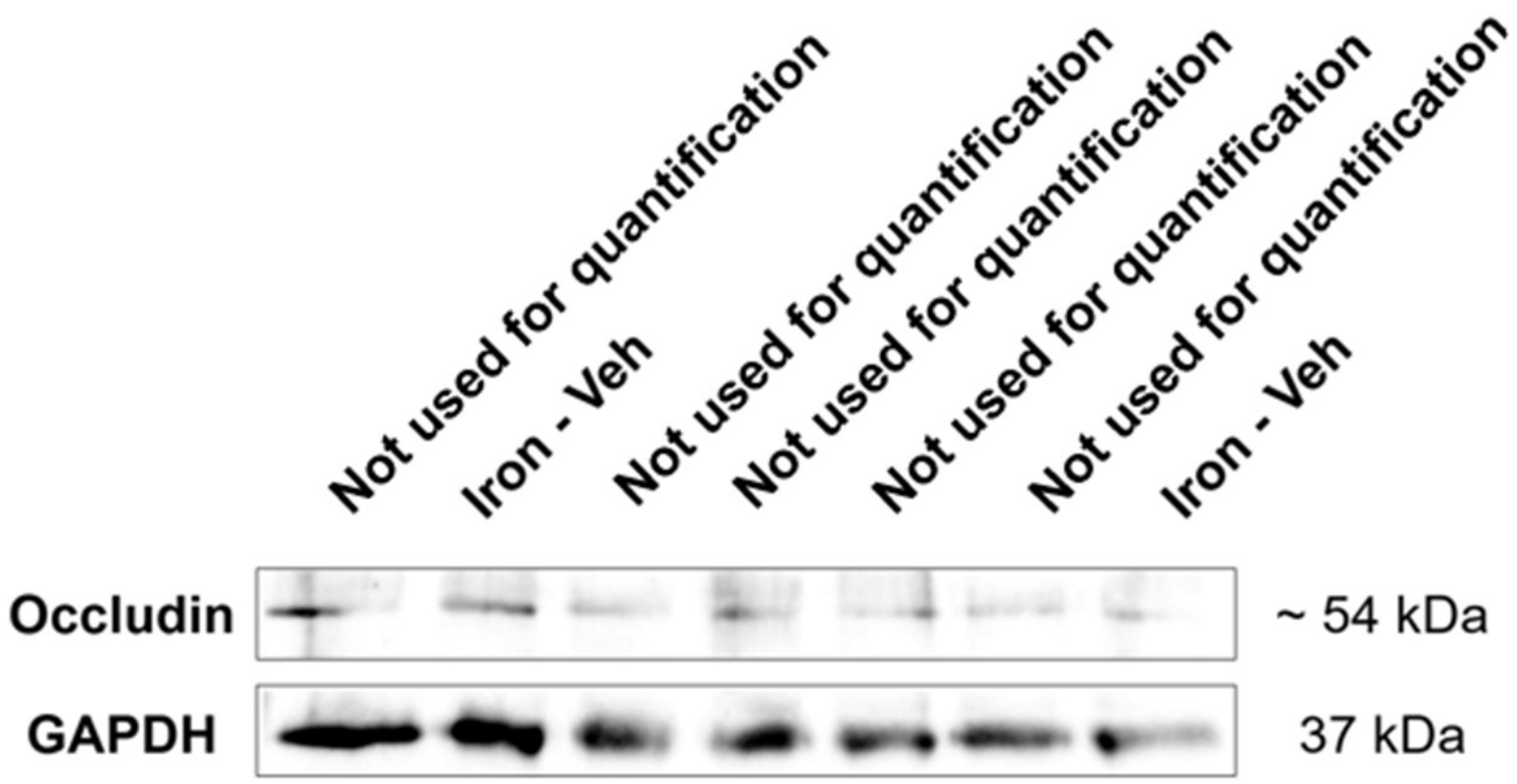

Exposure optimized for GAPDH

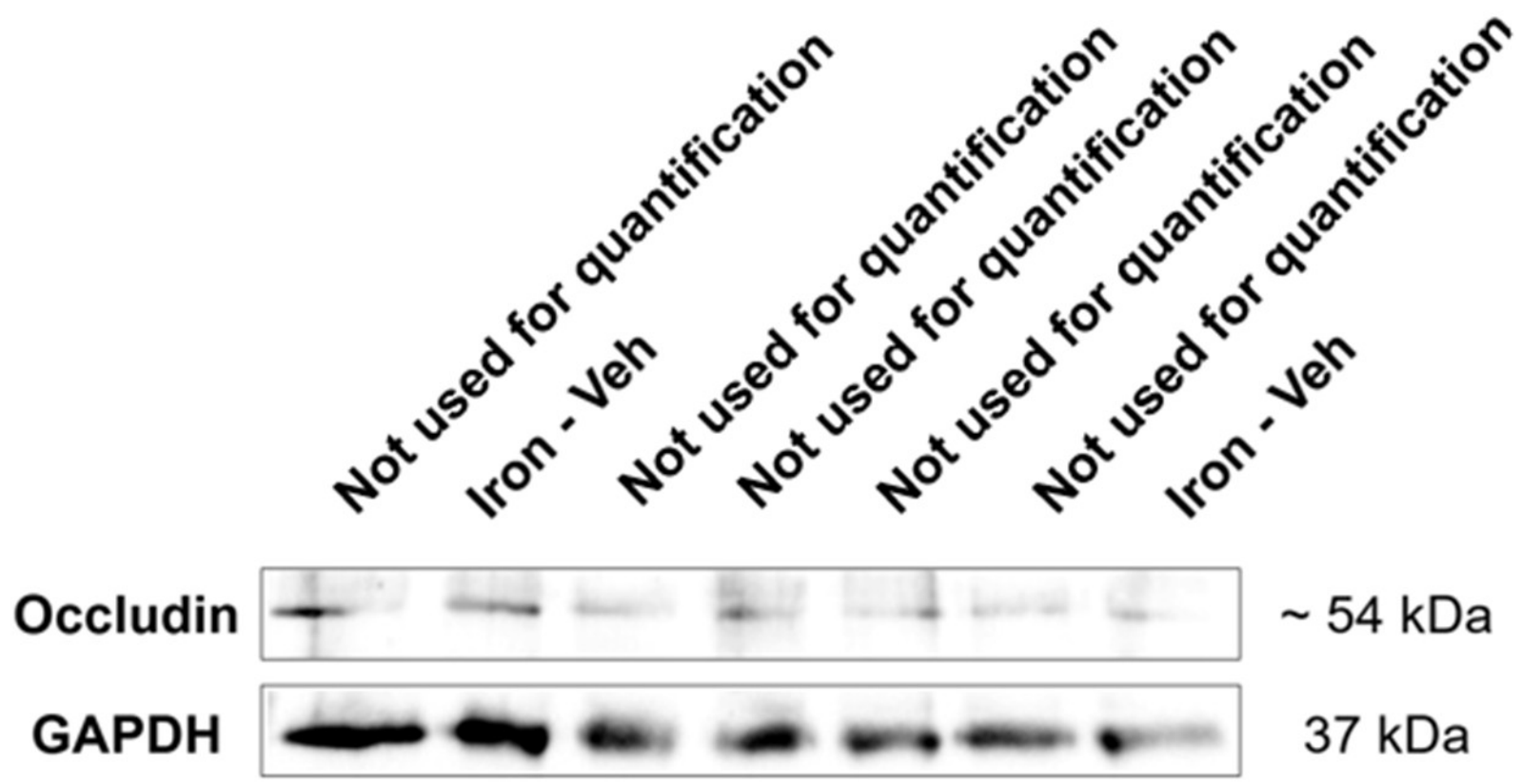

Exposure optimized for Occludin

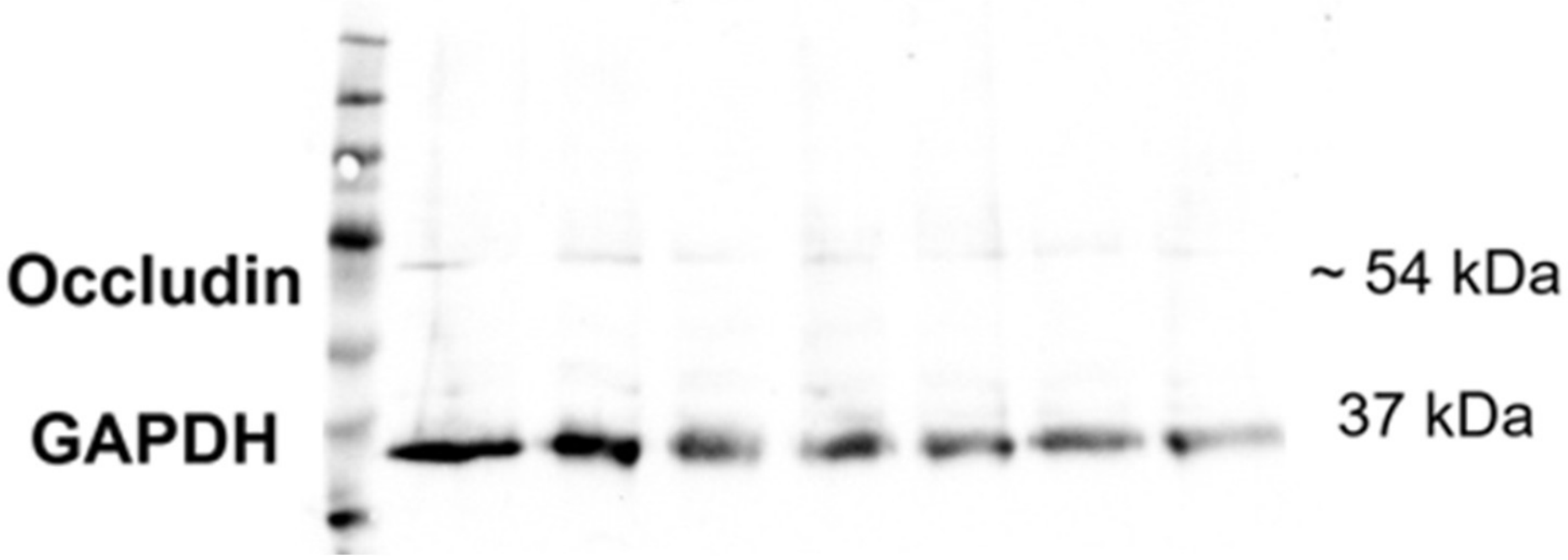

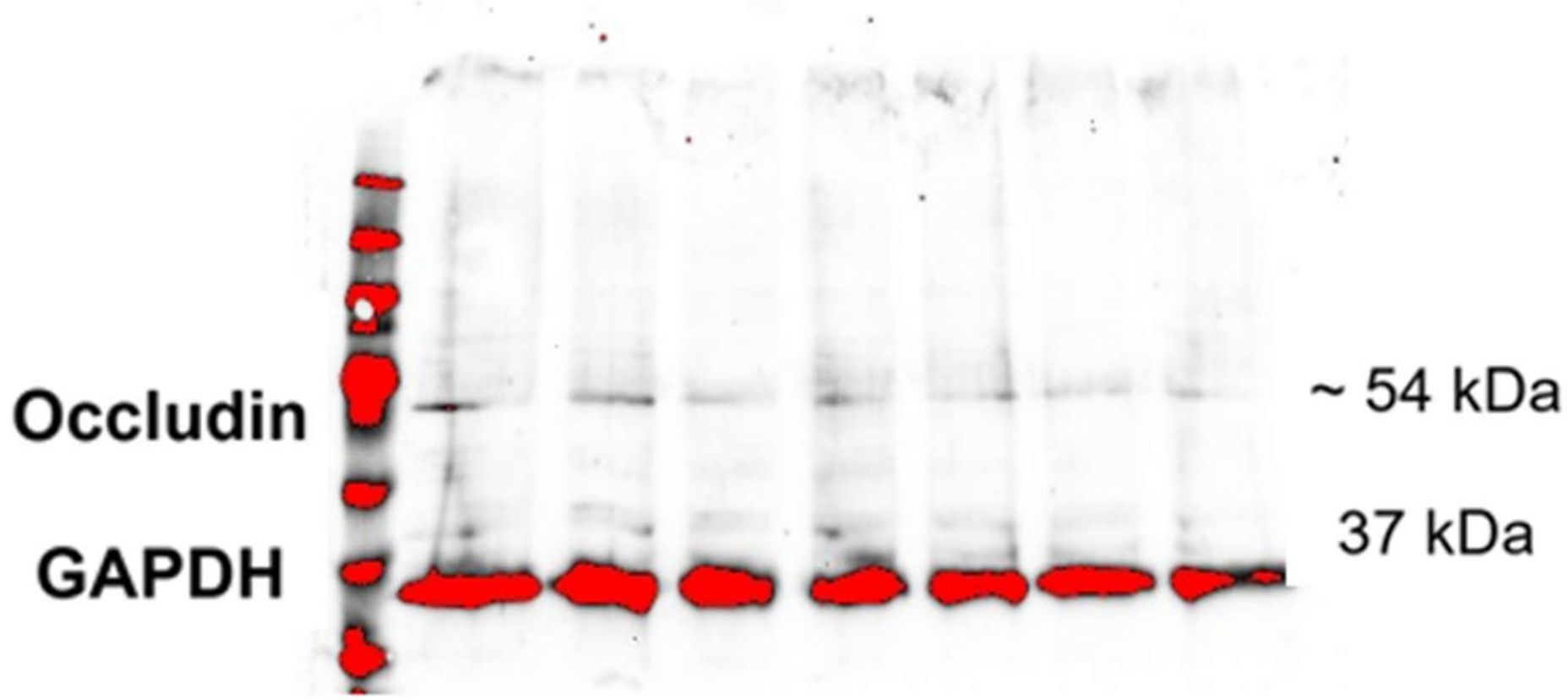

GEL 6

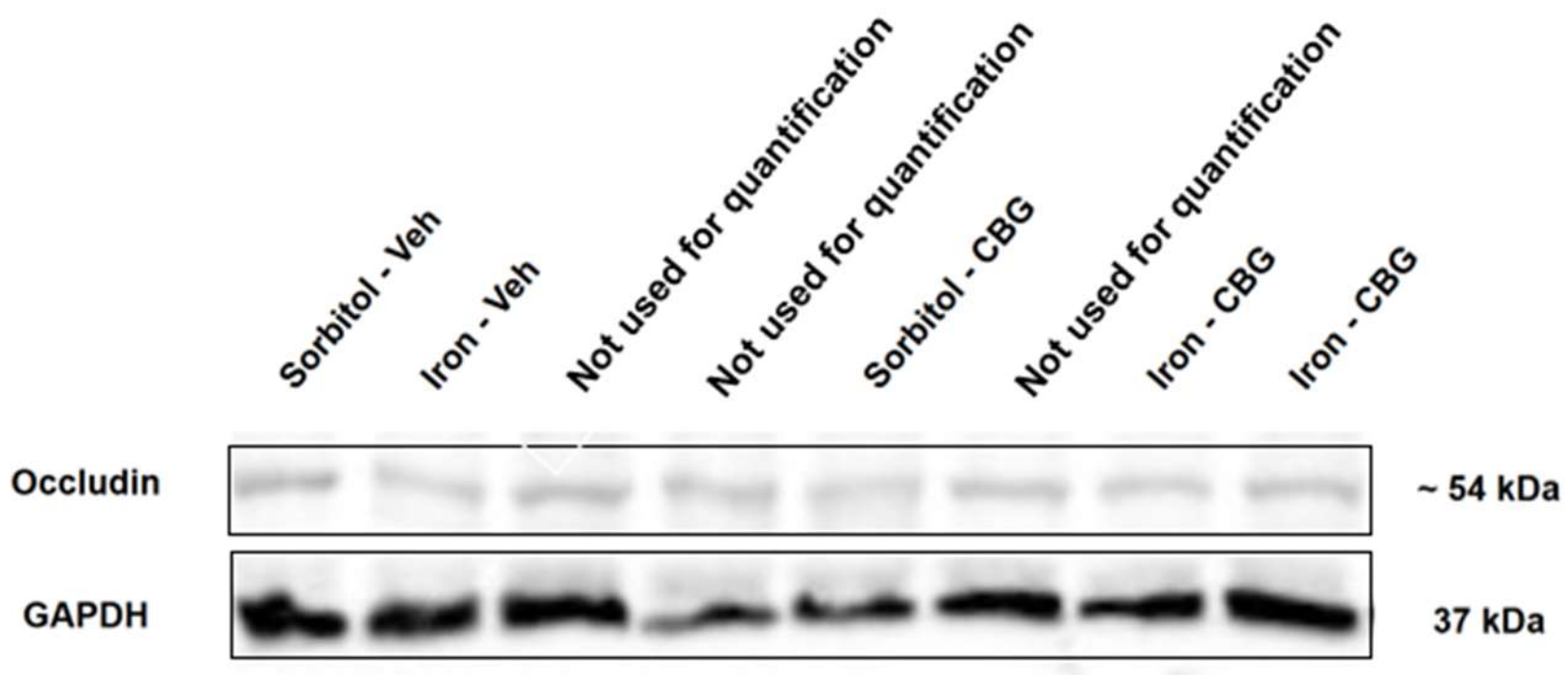

Exposure optimized for GAPDH

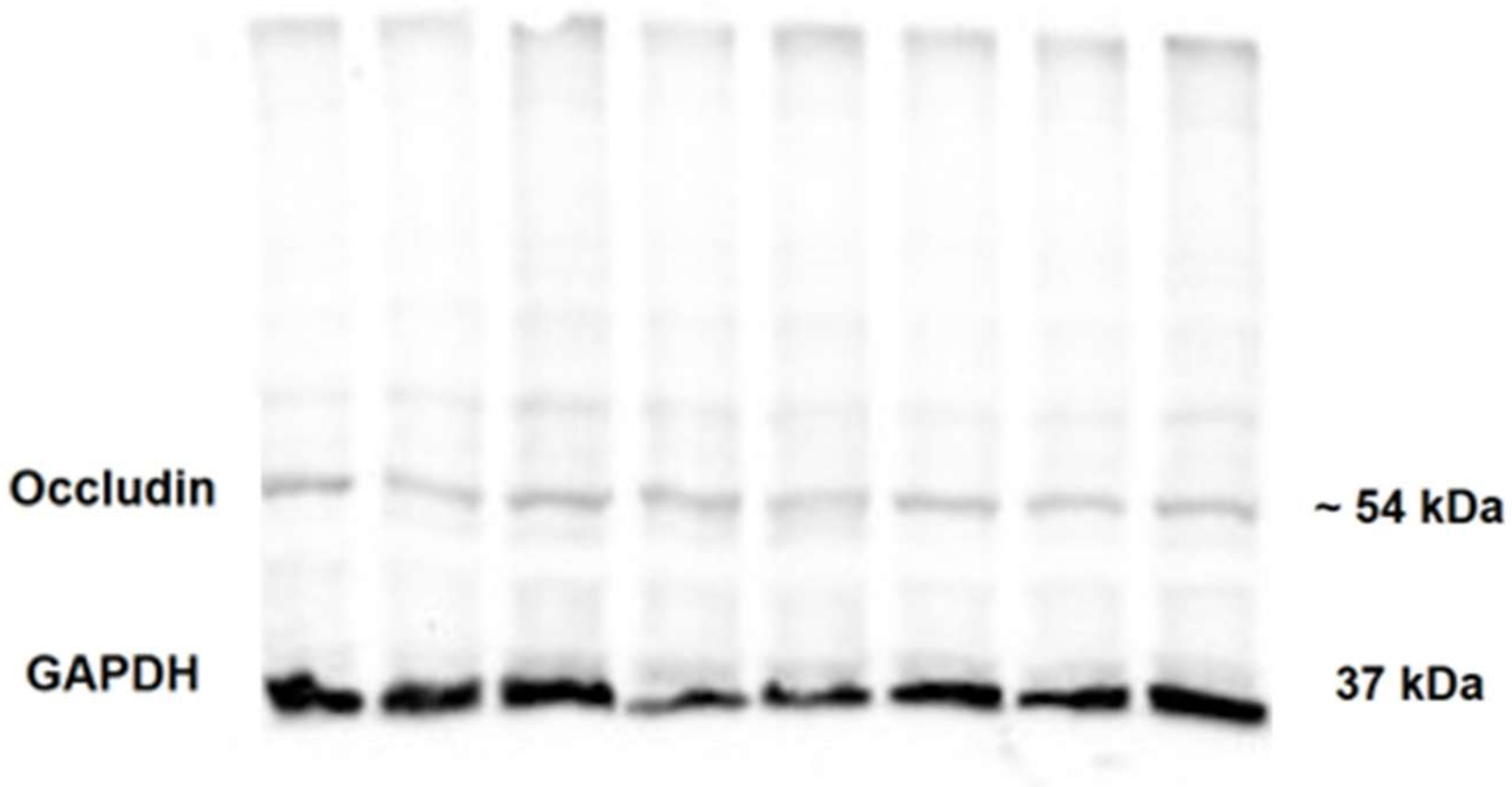

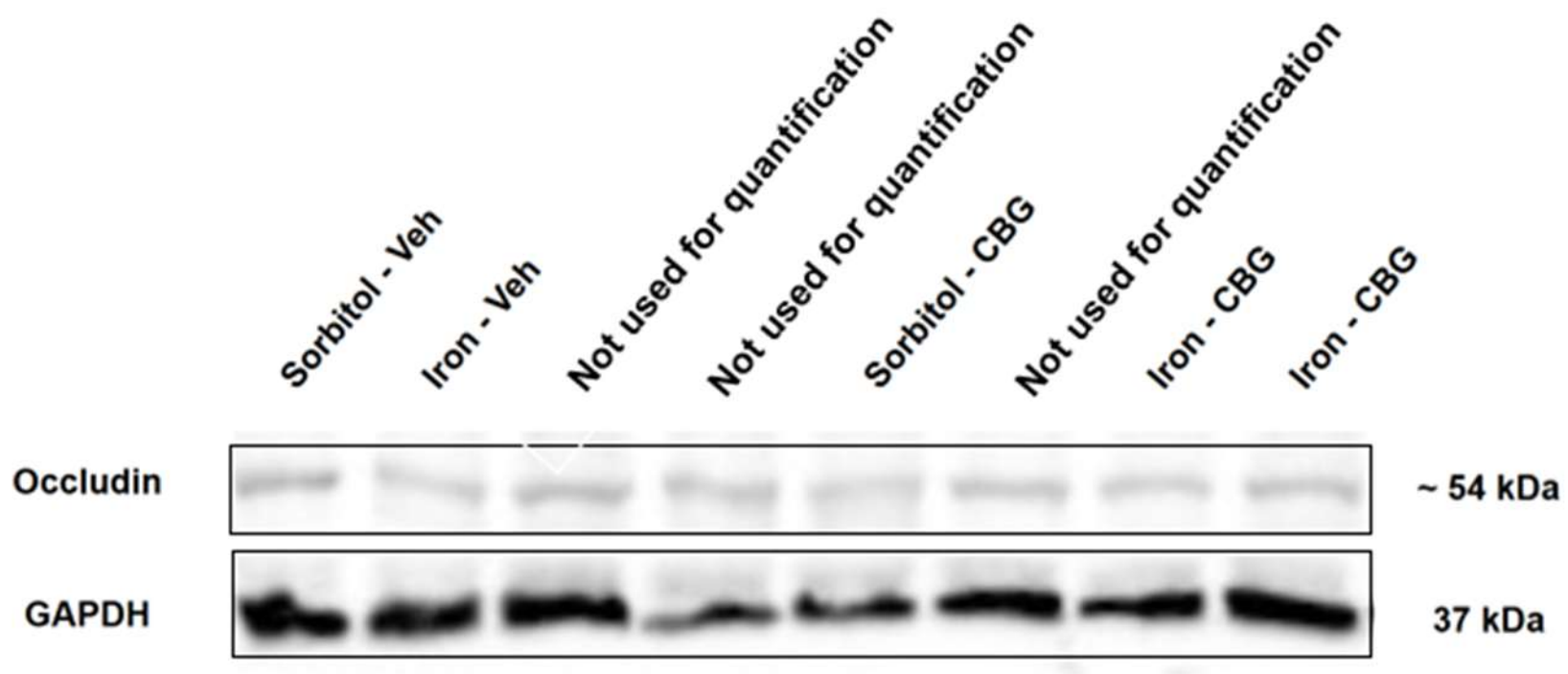

Exposure optimized for Occludin

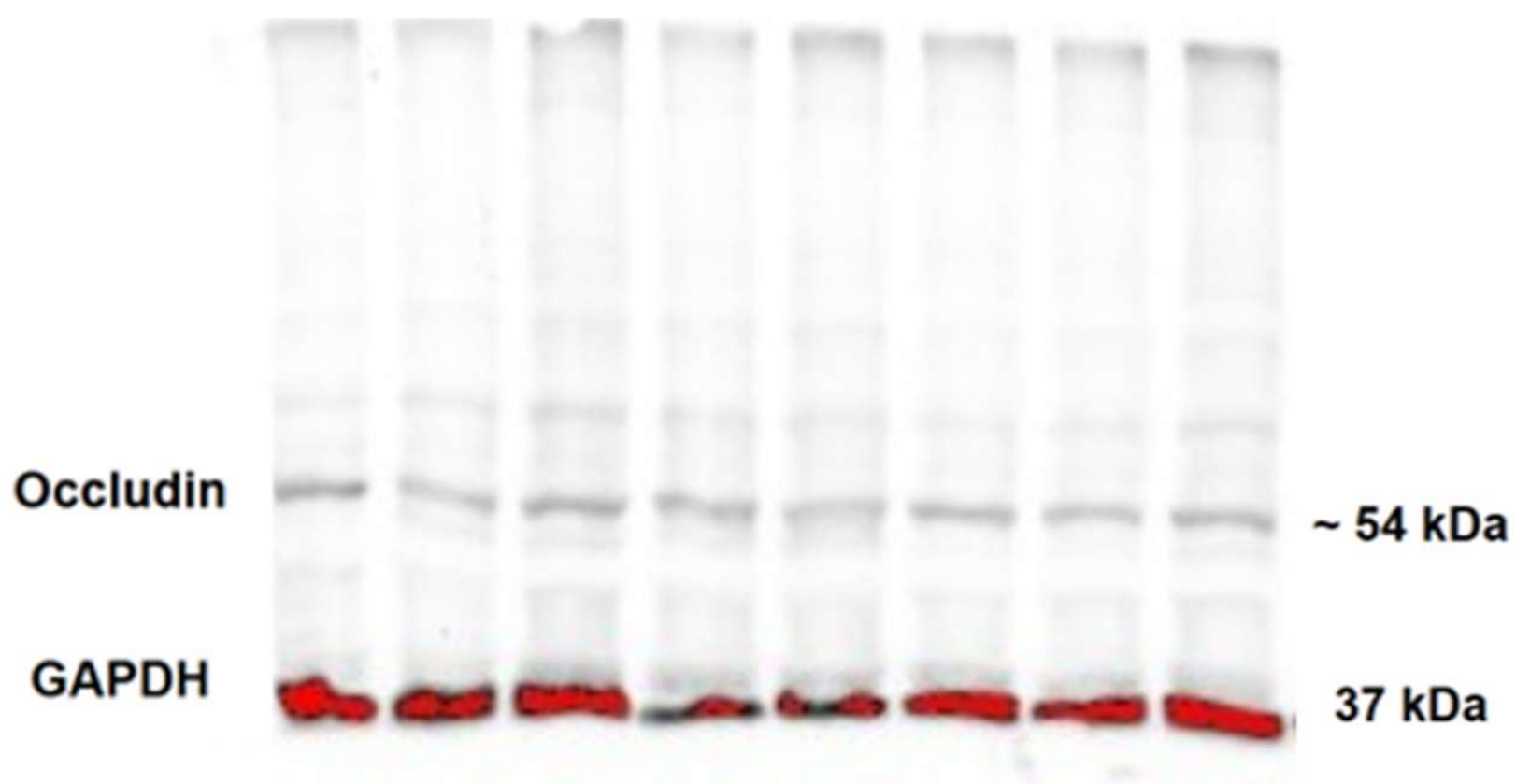

Supplement: Supplementary file 3 [file 12640_2026_826_MOESM3_ESM.pdf]
